# A prospective national precision medicine trial evaluating the feasibility of blood-based molecular profiling in patients with Cancer of Unknown Primary (CUP-COMP)

**DOI:** 10.1038/s41416-026-03519-6

**Published:** 2026-07-20

**Authors:** Alicia-Marie Conway, Matthew Robinson, Matthew Concannon, Sally Clive, Tania Tillett, Kai-Keen Shiu, Louise Medley, Sonali Dasgupta, Eliyaz Ahmed, Kelly Warrington, Usman Mahmood, Zoulikha Zair, Tess Gillham, Aaron Davis, Julie-Anne Scott, Alison Taylor, Mark Stares, Claire Mitchell, Pedro Oliviera, George J. Burghel, Natalie Cook

**Affiliations:** 1 Division of Cancer Sciences, Faculty of Biology, Medicine and Health, The University of Manchester, Manchester, UK; 2 Cancer Research UK National Biomarker Centre, The University of Manchester, Manchester, UK; 3 The Christie NHS Foundation Trust, Manchester, UK; 4 Faculty of Biology, Medicine and Health, The University of Manchester, Manchester, UK; 5 Edinburgh Cancer Centre, The University of Edinburgh, Western General Hospital, Edinburgh, UK; 6 Royal United Hospitals Bath NHS Foundation Trust, Bath, UK; 7 University College London & University College London Hospitals NHS Foundation Trust, London, UK; 8 Torbay and South Devon NHS Foundation Trust, Torquay, UK; 9 Velindre University NHS Trust, Cardiff, UK; 10 The Clatterbridge Cancer Centre, Liverpool, UK; 11 Roche Products Limited, Welwyn Garden City, UK; 12 Northern Ireland Cancer Centre, Belfast, UK; 13 Manchester Centre for Genomic Medicine and North West genomic laboratory Hub, Manchester University Hospitals NHS Foundation Trust, Manchester, UK; 14 Division of Evolution, Infection, and Genomic Sciences, The University of Manchester, Manchester, UK

**Keywords:** Predictive markers, Cancer genomics

## Abstract

**Background:**

Cancer of Unknown Primary (CUP) is an aggressive malignancy with poor patient outcomes. For most patients, the primary site remains elusive, limiting therapeutic options to non-specific chemotherapy. The CUP-COMP study (NCT04750109) assessed the feasibility of integrating molecular profiling with a National Molecular Tumour Board (MTB) into the treatment pathway of patients with CUP.

**Methods:**

Patients with histologically confirmed CUP were recruited from seven UK sites. Blood- and/or tissue-based molecular profiling was performed with results interpreted via an MTB identifying potentially actionable alterations (PAA). Retrospective tissue and blood alteration analysis assessed concordance and utility.

**Results:**

113/117 patients recruited between June 2021 and February 2023 completed 12 months of follow-up. Of 115 patients with successful molecular profiling, blood-based profiling was more successful (93%) than tissue-based (61%). The MTB identified PAAs in 63% (73/115), with 26% (31/117) treated using a precision medicine (PM) approach. Primary tumours were subsequently confirmed/suspected in 41 patients. Median overall survival (OS) was 9.5 months. Detecting a PAA in blood was impacted by tumour fraction (33% of patients had a tumour fraction <1%).

**Discussion:**

CUP-COMP demonstrates liquid biopsies are highly feasible in CUP. While tissue profiling remains preferable, blood-based profiling offers a timely viable alternative to tissue, especially in selected patients.

## Introduction

Cancer of Unknown Primary (CUP) represents a heterogeneous group of aggressive cancers where the origins of the cancer remain elusive. CUP accounts for 2–3% of all new cancer diagnoses but remains a leading cause of cancer death [1, 2]. Previously an under-researched entity, several recent studies have highlighted the importance of adequate diagnostic workup and the use of molecular profiling for diagnosis prediction and/or biomarker treatment stratification, including recently updated European Society for Medical Oncology (ESMO) guidelines [3–6]. Improved management pathways attempt to aid the diagnosis of otherwise ‘diagnostically challenging’ primary tumours that can often be misclassified as CUP and expand the classification of ‘favourable’ CUP subsets to include those tumours clinically aligned to known tumour types [3]. Patients within these ‘favourable’ subsets (~20%) can access the aligned tumour site-directed therapies and achieve better clinical outcomes [7]. However, the majority of patients (80–85%) fall within the ‘unfavourable’ or ‘poor-risk’ CUP subset where treatment is limited to ‘one-size-fits-all’ chemotherapy with poor efficacy and resulting dismal prognosis of less than 1 year [2]. The recently reported CUPISCO trial has demonstrated molecular profiling has clinical utility for improving treatment stratification and outcomes in patients with CUP [4], however clinical feasibility of the approach, including molecular profiling modality selection (tissue vs. blood, whole genome sequencing vs. large panel sequencing), mechanisms for interpretation of results and utility in broader CUP cohorts remain unexplored. CUP-COMP was an investigator-initiated multi-centre prospective non-randomised clinical trial comparing the clinical feasibility and utility of molecular profiling from tissue and blood and the integration of a National Molecular Tumour Board into the UK CUP management pathway. To overcome the known challenges of accessing good quality tissue for profiling in this population we compare the clinical utility of tissue and blood-based circulating tumour DNA (ctDNA) profiling.

## Methods

### Trial design and ethical approval

CUP-COMP (clinicaltrials.gov registration ID: NCT04750109) was conducted in accordance with Good Clinical Practice and the ethical principles originating from the Declaration of Helsinki. Ethical approval was obtained from the Yorkshire and The Humber–Sheffield Research Ethics Committee (REC: 20/YH/0305) and trial sponsorship was undertaken by The Christie NHS Foundation Trust. The primary objective of the study was to establish a mechanism to assess, compare and interpret molecular profiling results (from tissue and blood) for the diagnosis and treatment stratification for patients with CUP (for detailed primary and secondary outcome measures see Supplementary Methods). All patients provided fully informed written consent for the trial with optional consent regarding genetic result disclosure and acquisition of additional research samples (Supplementary Methods).

### Patient recruitment and sample collection

Patients with CUP were recruited between June 2021 and February 2023 through seven United Kingdom sites (Supplementary Methods). Eligibility was determined locally, and inclusion criteria included: appropriate local pathological confirmation of a diagnosis of ‘favourable’ or ‘poor-risk’ CUP according to ESMO guidelines 2015 [8] and discussion at a local multi-disciplinary team meeting (MDT). Patients were required to have an Eastern Cooperative Oncology Group performance status (ECOG PS) of 0–2 and were eligible if treatment-naïve or previously treated. As this was a feasibility study no sample size calculation was performed and sample size was determined by study duration and funding allocation for molecular profiling. At the time of trial design only select single gene molecular testing on tissue was available in the UK for patients with CUP, with approval for Whole Genome Sequencing (WGS) introduced by NHS genomic Hubs in January 2022, with the requirement for fresh tissue biopsy.

### Genomic analysis

All consenting patients provided baseline blood samples for genomic profiling using the FoundationOne®Liquid CDx (F1LCDx) assay (Foundation Medicine, Inc). After trial amendments were made, diagnostic formalin fixed paraffin-embedded (FFPE) tissue were retrospectively profiled using the FoundationOne® CDx (F1CDx) assay (Foundation Medicine, Inc) where suitable residual tissue was available. Where clinically appropriate, patients had additional blood draws on progression following treatment for additional F1LCDx testing. Patients consenting to a fresh tissue biopsy also provided samples for WGS and an additional blood sample for germline control. DNA extraction of germline blood and fresh frozen tissue was performed by The North West Genomic Laboratory Hub (NW GLH), Manchester and WGS was performed by Source Biosciences UK Ltd (Nottingham, UK) (Molecular profiling details are supplied in Supplementary Methods).

### Molecular Tumour Board (MTB)

A monthly virtual CUP MTB, with an integrated digital platform for genomic analysis interpretation (eTARGET) (https://upsmart.digitalecmt.com/etarget-3/), was attended by treating medical oncologists, site-based healthcare professionals, and clinical scientists from the NW GLH. Variant interpretation was undertaken by clinical scientists from the NW GLH including: classification for pathogenicity/oncogenicity assigned according to relevant guidelines [9–12] and identification of variants of potential germline origins according to ESMO guidance [13], including assessment of variants suspected to be associated with Clonal Haematopoiesis of Indeterminate Potential (CHIP). Variant interpretation results were discussed in the context of clinical cases, including patterns of alterations that indicated a primary tumour origin, and those alterations that may be predictive of response to an existing Standard of Care (SoC) or experimental therapy (potentially actionable alteration; PAA). The potential actionability of alterations was determined by the research team based on available evidence at the time and considered potential pre-clinical evidence and knowledge of early phase trials open within the UK (using a trial matching tool within eTARGET). Recommendations for treatment, trial entry or further confirmatory testing were communicated to the treating team, including recommendations for clinical genetic referrals.

### Data collection and analysis

Patients’ clinical data was collected at 6- and 12-months following recruitment to study including: receipt of any systemic anti-cancer therapy (SACT), radiotherapy or entry into a clinical trial, if a primary tumour diagnosis was determined/suspected, survival status, date of last clinical contact, and date of death. Scottish Inflammatory Prognostic Score (SIPS) was calculated where baseline blood tests results were available, as previously described [14]. MTB outcome data was extracted from electronically recorded outcomes.

### Statistical analysis and post-hoc survival analysis

Median overall survival (OS), with associated 95% confidence intervals (CI), was estimated using the Kaplan-Meier method, from the date of consent until 12-month follow-up for all patients with complete follow-up data (*n* = 113). To compare survival by treatment, post-hoc groupings were defined according to interventions received by patients after consent. Group 1 patients were defined as: those patients that received doublet-platinum based chemotherapy (Doublet Chemotherapy) that would be SoC for CUP, or single agent paclitaxel in the second line setting. Group 2 patients were defined as those patients that did not receive any systemic anticancer therapy (SACT) or were treated with Best Supportive Care (BSC). Group 3 patients were defined as those patients receiving a Precision Medicine (PM) approach: they received either a targeted therapy or immunotherapy through trial, SoC, or compassionate access and/or received treatment specific for a subsequently determined tumour type. Of note, patients that received doublet chemotherapy after a confirmed or suspected primary site determined were defined as Group 3 as the chemotherapy regimen was determined to be site-directed. All patients with follow-up data were included, except patients with a neuroendocrine carcinoma (NEC) histology, due to subsequent exclusion of NEC as a CUP subtype from the updated 2023 ESMO CUP guidelines, and patients treated radically with surgical resection. Differences between the groups were assessed using a pairwise Mantel-Cox Log-rank test to assess significance and hazard ratios, with *p*-values derived from two-sided tests.

A univariate Cox-proportional hazard model was used to assess the association between comprehensive tumour fraction score (CTFS; see Supplementary Methods) and survival. Clinical characteristics, including CTFS and SIPS, were compared between the three treatment groups using Chi-squared, Fisher’s exact two-sided tests, or the Kruskal-Wallis rank sum test. A CTFS of <1% was used to define low tumour fraction for comparisons. All univariate and comparison statistical analyses were performed using SPSS version 29.0.0.0 and GraphPad Prism version 10. Survival analyses were performed using RStudio (version 2024.04.2), using standard packages. The analysis code is available from the corresponding author upon reasonable request. A two-sided significance level of 5% was used throughout.

### Retrospective genomic analysis

Retrospective analysis of all genomic data were performed to assess pathogenicity utilising genomic interpretation software (OncoKB) and to compare tumour and blood alteration concordance (Supplementary Methods).

## Results

**Fig. 1:**
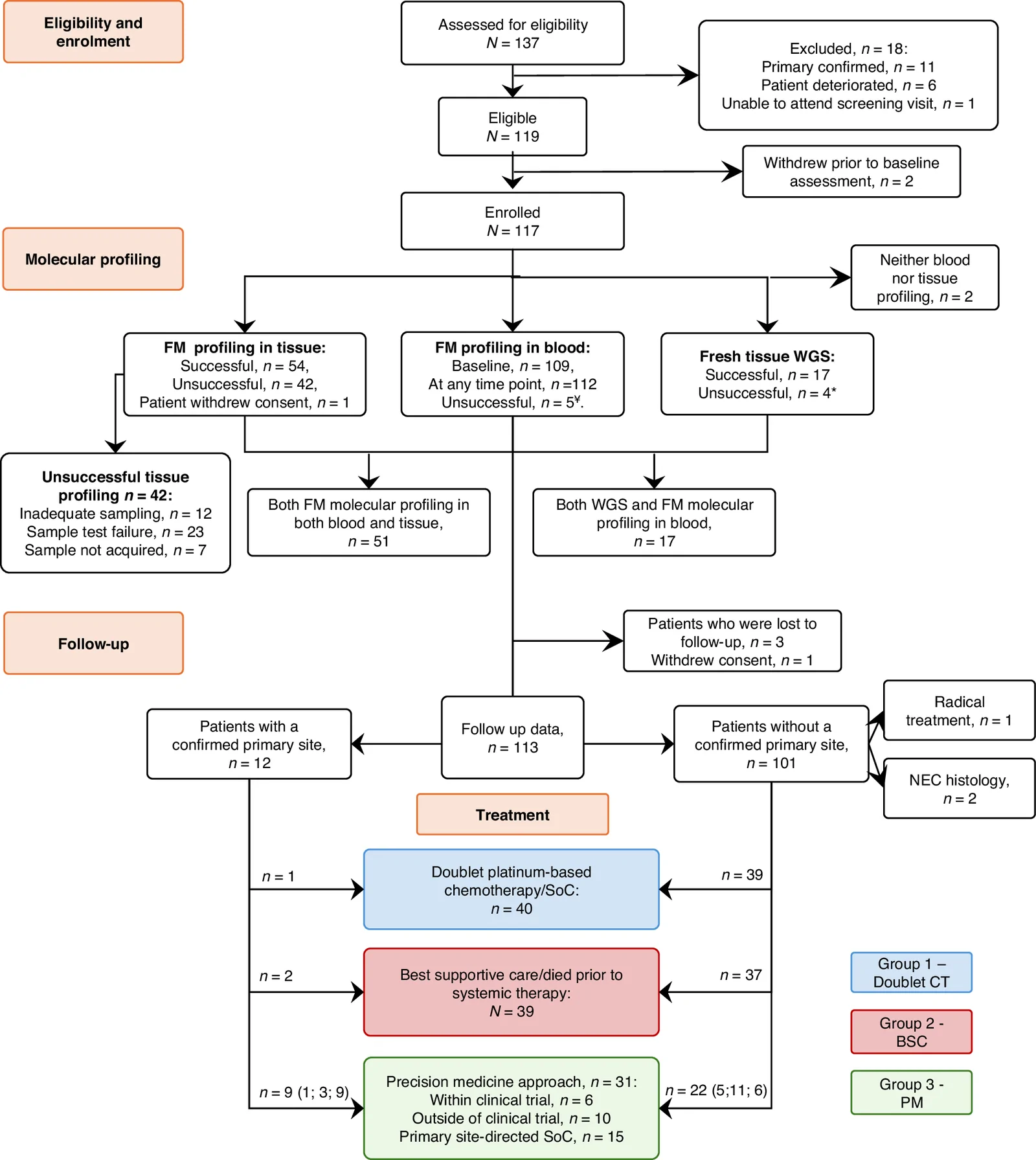
Consort diagram. Final treatment groupings shown in coloured box; Blue = Group 1 received standard of care (SoC) doublet platinum-based chemotherapy (Doublet CT); Red = Group 2 patients that died prior to systemic therapy or received best supportive care (BSC); Green = Group 3 patients that received a precision medicine (PM) approach. ^¥^ Insufficient blood draw/DNA yield/patient already commenced chemotherapy *sample mishandling.

Of 137 patients assessed for eligibility, 117 patients with a histologically confirmed diagnosis of CUP were enroled (Fig. 1). The baseline demographics of the enroled participants are shown in Table 1. The median age was 63 years, and 66 (56%) participants were female. At baseline, the ECOG PS was 0 or 1 in 95 (81%) participants and 64 (55%) were never smokers. Histological subtypes included adenocarcinoma (*n* = 62, 53%), carcinoma (*n* = 29, 25%) and squamous cell carcinoma (*n* = 19, 16%). Differentiation was recorded for only 65 (55%) patients with 52 of these recorded as poorly differentiated (*n* = 50) or undifferentiated (*n* = 2) (Table 1). Thirty-six patients (31%) had metastatic disease restricted to a single organ site. Two patients had NEC histology and were excluded from downstream survival analysis following the 2023 ESMO CUP guidelines, which excludes NEC as a subtype of CUP. Thirty-six patients (31%) had received at least one prior line of therapy before consent; the remaining 81 patients were treatment naïve.

**Table 1:** Baseline participant demographics.

Characteristic		*n* = 117 (%)
Age at consent		63 [54, 72]
Gender	Female	66 (56%)
	Male	51 (44%)
Ethnicity	African	1 (0.9%)
	Any other Asian background	1 (0.9%)
	Any other white background	8 (6.8%)
	British	90 (77%)
	Chinese	1 (0.9%)
	Missing	1 (0.9%)
	Unknown	15 (13%)
Histological type	Adenocarcinoma	62 (53%)
	Carcinoma	29 (25%)
	Other	4 (3.4%)
	Squamous Carcinoma	19 (16%)
	Unknown	3 (2.6%)
Degree of differentiation	Well	4 (3.4%)
	Moderate	9 (7.7%)
	Poor	50 (43%)
	Undifferentiated	2 (1.7%)
	Not specified	47 (40%)
	Missing	5 (4.3%)
Median number of metastatic sites (IQR)		3 (2, 4)
Metastasis to bone		33 (29%)
Metastasis to the liver		29 (25%)
Metastasis to the lymph nodes		70 (60%)
Weight Loss in last 6 months?	Yes	34 (29%)
	No	74 (63%)
	Missing	9 (7.7%)
ECOG PS	0	35 (30%)
	1	60 (51%)
	2 +	20 (17%)
	Unknown	2 (1.7%)
Smoking Status	Current	19 (16%)
	Missing	1 (0.9%)
	Never	64 (55%)
	Past	31 (26%)
	Unknown	2 (1.7%)
Family history of cancer	No	31 (26%)
	Yes	49 (42%)
	Unknown	37 (32%)

*ECOG* PS Eastern Cooperative Oncology Group Performance Status; *IQR* Interquartile Range.

### Blood-based molecular profiling was more clinically feasible than tissue-based molecular profiling

**Fig. 2:**
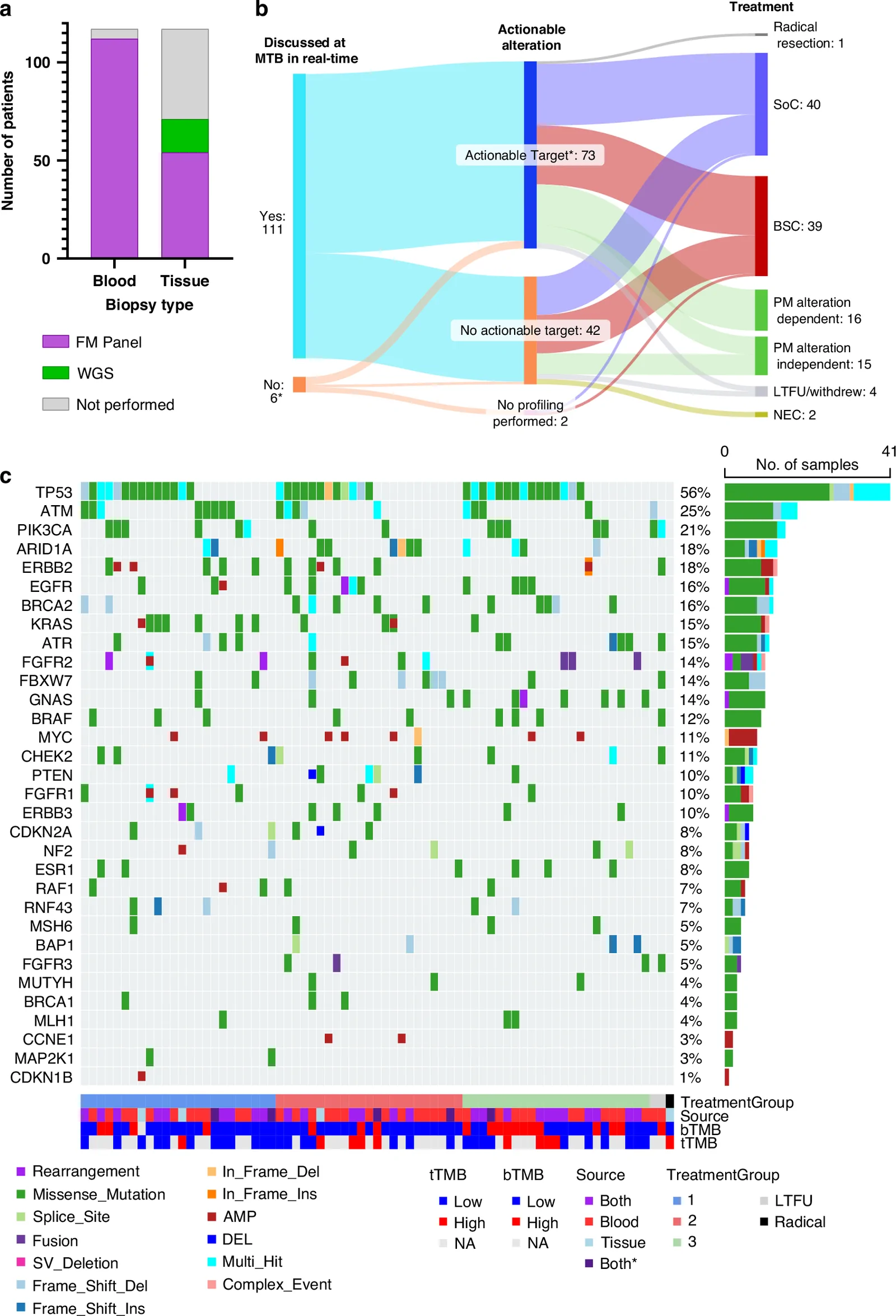
Real-time molecular data interpretation and summary. **a** Successful molecular profiling in blood and tissue; *two patients had neither blood nor tissue profiling performed. **b** Sankey plot of Molecular Tumour Board (MTB) outcome discussions linked to treatment outcomes. Colour codes of treatment represent final treatment groupings. (Blue = Group 1: Standard of Care Doublet Chemotherapy (SoC); Red = Group 2: Best supportive care (BSC)/No treatment; Green = Group 3: Precision Medicine (PM) approach) *4 patients results reviewed in retrospective MTB and 3 determined potentially actionable alterations. **c** Oncoplot of select genes highlighted in MTB discussions as potentially actionable in 73 patients. Blood and tissue Tumour Mutation Burden (bTMB and tTMB) status annotated, and source corresponds to which profiling source the alteration was found in. Both * means partial concordance where more than one potentially actionable alteration found. FM Foundation Medicine, LTFU Lost to follow up NEC Neuroendocrine Carcinoma, WGS Whole genome sequencing.

Molecular profiling via tissue and/or blood was successful at some point during trial enrolment in 115/117 patients (Figs. 1, 2, summarised in Supplementary Table 1 and detailed by patient in Supplementary Table 2). In the 2 patients where molecular profiling was not achieved, blood-based profiling failed due to insufficient DNA yield or insufficient blood draw for testing. At baseline, 109/117 (93%) patients had successful blood profiling (Fig. 2a).

Originally 21/117 (18%) patients consented to a fresh biopsy sample to be taken, and 17 patients had successful WGS performed. There were significant issues and delays to recruitment due to the mandated trial biopsy, therefore a trial amendment was introduced for optional fresh biopsy, and the remaining consenting cohort (95/117, excluding one patient who withdrew consent for the study prior to tissue testing) had diagnostic FFPE tissue assessed for F1CDx testing resulting in 54/95 (57%) cases with successful tissue profiling (Figs. 1, 2 and Supplementary Table 1). Including progression blood samples, 68 patients had both tissue and blood molecular profiling performed during study: 17 with WGS and F1LCDx blood and 51 with F1LCDx blood and F1CDx tissue. Feasibility of the three profiling approaches, including success of profiling and failure reasons are detailed in Supplementary Table 1.

### Real-time clinical decision-making at the National Molecular Tumour Board

Real-time molecular profiling interpretation and MTB discussions occurred for 111/117 (95%) patients (Fig. 2b). For blood profiling 109/117 (93%) patients had real-time MTB discussions where time from baseline Blood T1FM report received until MTB discussion could be calculated. Of those patients discussed 74/109 (68%) were discussed within 31 days of report and 35/109 (32%) had an MTB discussion >31 days from report date. Where MTB discussions took place >31 days from report the central or local site determined delay to discussion was appropriate either due to no alterations found on profiling or discussion was no longer clinically required due to patient deterioration. For 4 patients’ molecular results were reported but not discussed in real-time in the MTB, reason unknown (Fig. 2b). Excluding the 4 patients that were discussed retrospectively, the median time to discussion was 20 days (range 5–164 days). Turnaround time of baseline F1LCDx blood testing (*n* = 109) from blood draw to report was on average 15 days (range 8–34 days).

MTB-determined PAAs were found in 73/115 (63%) patients with successful blood and/or tissue molecular profiling performed, including 3 patients discussed retrospectively (Fig. 2b, c). The median number of PAAs per patient was 1 (range 1–6), with 24/73 (33%) patients harbouring more than one PAA. For 38/73 (52%) of these patients, the PAA was found in blood alone (only 6 of these patients also had tissue profiling), 29/73 (40%) the PAA was found in both modalities and 4 patients had PAAs found only in tissue profiling (with 3/4 of these patients having blood profiling performed) (Fig. 2c).

Across the whole cohort (*n* = 117), 31 (26%) patients were treated with a precision medicine (PM) approach (Group 3, see methods): 6 patients were treated within a clinical trial, 10 patients accessed immunotherapy outside of a clinical trial, and 15 patients had SoC treatment directed to a confirmed/suspected primary tumour site (Fig. 1 and Supplementary Table 2). Within the PM group (*n* = 31), 16 patients had treatment targeting a specific PAA identified through molecular profiling (16/117; 14% of total cohort). Nine patients of the total cohort (9/117; 8%) harboured suspected pathogenic germline variants and were discussed at a germline MTB for specialist genetics input (Supplementary Table 2).

### Clinical outcomes and survival analysis

**Fig. 3:**
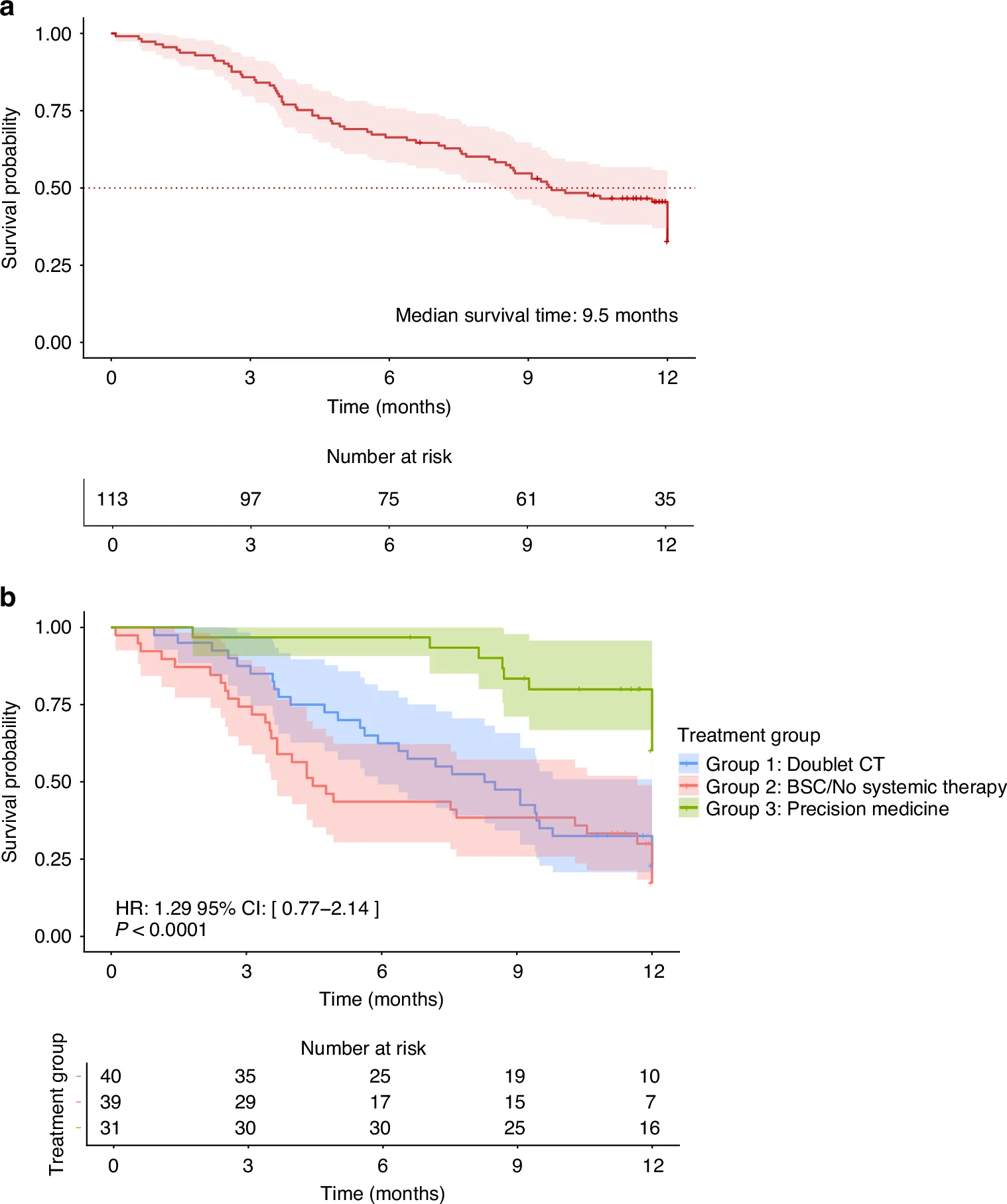
Survival analysis. **a** Whole cohort with sufficient follow up data (*n* = 113). **b** Survival analysis split by post-hoc treatment categorisation (*n* = 110); excluding patients with neuroendocrine histology (*n* = 2) and patient who underwent radical resection of oligometastasis (*n* = 1). BSC Best Supportive Care, CI Confidence Intervals, Doublet CT Doublet chemotherapy, HR hazard Ratio, PM Precision Medicine.

Of the 117 patients enroled, 113 completed the 12-month follow-up (*n* = 3 lost to follow-up; *n* = 1 withdrew consent) (Fig. 1). Forty-one (35%) patients had a primary tumour diagnosis confirmed or clinically suspected during the study period (12 confirmed; 29 suspected) (Fig. 1 and Supplementary Fig. 1). For 8 patients, molecular alterations led to or supported a clinical suspicion of a primary tumour type (Supplementary Fig. 1; Supplementary Table 2). In addition, for 3 patients with a confirmed iCCA diagnosis, molecular profiling on study supported this diagnosis (all 3 had FGFR2-fusions found on profiling) and enabled access to appropriate SoC therapies including targeted therapies. The median OS for the follow-up cohort (*n* = 113) was 9.5 months (Fig. 3a).

Post-hoc analysis based on treatment following consent to trial was performed on 110 patients where survival data was available, excluding those patients treated radically with surgery (*n* = 1) or with NEC (*n* = 2) (see Methods). 69 of 110 (63%) patients of this cohort went on to have SACT and 41 of 110 (37%) were treated with best supportive care (BSC) (Fig. 1).

OS was significantly longer in patients treated with a PM approach (Group 3) when compared to patients treated with SoC Doublet chemotherapy (Group 1); median OS was not reached in Group 3 vs 8.4 months in Group 1 (HR 0.29 [CI 0.14–0.59], *p* < 0.0001) (Fig. 3b). To further elucidate the contribution of a biomarker-driven approach vs a change in diagnosis, survival analysis within the PM Group 3 was performed. There was no significant difference in survival between those patients treated based on a change in diagnosis/suspected diagnosis (*n* = 16, Group A) compared to those who received treatment directed to a PAA (*n* = 12, Group B) (Supplementary Fig. 2, HR 0.28 95% CI: 0.06–1.31, *p* = 0.083, excludes *n* = 3 patients). There was no statistical difference in survival between patients treated with Doublet chemotherapy (Group 1) vs those that received BSC (Group 2); median OS was 8.4 and 4.5 months, respectively (HR 1.29 [CI 0.77–2.14], *p*-value 0.3317). Between the 3 treatment groups there was no significant difference in baseline characteristics except ECOG PS (Fisher exact test, two-sided, *p* = 0.019) and notably no difference in Scottish Inflammatory Prognostic Score (SIPS) (Supplementary Table 3).

Survival remained superior in the PM Group 3 when restricted to those patients with no prior SACT (*n* = 75) (Supplementary Fig. 3a, b). Median OS was not reached in the PM Group 3 compared to 8.5 months in the Doublet chemotherapy Group 1 (HR of 0.24 [CI 0.09–0.64], *p*-value 0.0042). In the treatment naïve setting, survival was significantly improved in the Doublet chemotherapy (Group 1) compared to BSC (Group 2; Median OS of 3.7, HR of 1.87 [CI 1.04–3.35], *p*-value 0.0367).

### Retrospective analysis of blood-based molecular profiling to evaluate clinical utility

To further assess the feasibility and clinical utility of blood-based molecular profiling, retrospective analysis was performed. Firstly, to compare the concordance of the blood and tissue approaches, the cohort was restricted to those patients where Foundation Medicine genomic profiling had been performed in both tissue and blood (*n* = 51). The median interval between tissue biopsy and blood draw was 59 days (average 142 days; range −307 days and +1651 days).

**Fig. 4:**
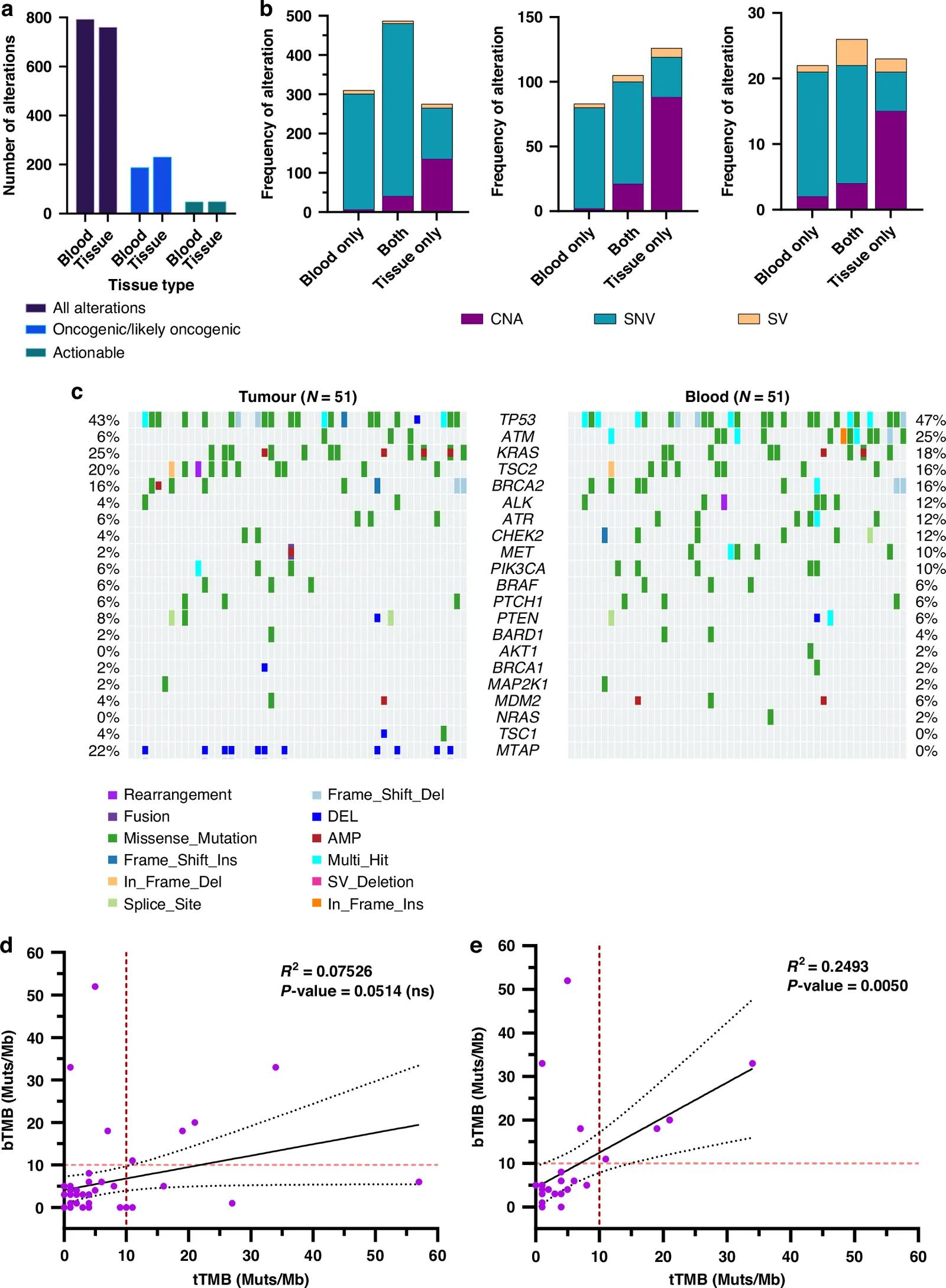
Retrospective comparison of tissue and blood molecular profiling (*n* = 51). **a** Number of alterations reported in blood and tissue: total number within cohort; number determined to be ‘Oncogenic’ or ‘Likely Oncogenic’ by OncoKB analysis; and number deemed actionable to level 3 evidence and above. **b** Frequency of alterations in blood and tissue by alteration pathogenecity and actionability (Left, All alterations; Middle, Oncogenic/Likely oncogenic; Right, Actionable), colour coded by alteration type. **c** Co-Oncoplot of alterations in genes determined to be potentially actionable by OncoKB analysis, plots split by profiling type (tissue or blood), column order is preserved across plots. **d** Comparison of Tumour Mutation Burden (TMB) in blood (bTMB) and tumour (tTMB) (*n* = 51). **e** Comparison of Tumour Mutation Burden (TMB) in blood (bTMB) and tumour (tTMB) excluding those samples with Comprehensive Tumour Fraction Score of (CTFS) < 1% (*n* = 30). Red dotted line at 10 Muts/Mb. AMP Amplification, CNA Copy Number Alterations, DEL deletion, Ins Insertion, Muts/Mb Mutations/Megabase, SNV Single Nucleotide Variants, SV Structural Variants.

Although the total number of reported alterations in blood and tissue was similar across the cohort (Fig. 4a, 1072 unique alterations), only 487 (45%) alterations were reported in both modalities (Supplementary Fig. 4A). A high proportion of blood-only alterations were Single Nucleotide Variants (SNV), whereas tissue-only alterations were more likely to be Copy Number Alterations (CNA), particularly deletions (Fig. 4b). This remained true when restricting to alterations that were predicted as oncogenic and to PAAs as determined by OncoKB (Fig. 4b, c and Supplementary Fig. 4A). On a patient level, concordance ranged from 9 to 89% (average 47%), and concordance rate was significantly correlated with CTFS (Pearsons’s correlation R^2^ = 0.1139, *p* = 0.0154; Supplementary Fig. 4B).

A Tumour Mutation Burden (TMB) of ≥10 Muts/Mb in either blood or tissue was reported in 23 patients: 10 patients in tissue only, 8 patients in blood only, 5 patients in both modalities. Where TMB was ≥10 Muts/Mb in tissue but not in blood (*n* = 5), the CTFS in blood was 0%. There was a non-significant correlation between blood-TMB (bTMB) and tissue-TMB (tTMB) across all 51 patients (Pearson correlation coefficient, R^2^ = 0.07526, *p* = 0.0514) but significant correlation when restricted to patients with CTFS of ≥1% (*n* = 30, Pearsons correlation, R^2^ = 0.2493, *p* = 0.0050; Fig. 4d, e).

To further explore the clinical utility of blood-based profiling in the absence of tissue, all patients with at least one blood sample were evaluated (*n* = 112). Sixty-five of 112 (58%) patients harboured at least one OncoKB-determined PAA (level 1-3B actionability) (Supplementary Fig. 5a–c). Level 1 evidence of actionability included twenty patients with a bTMB of ≥10 and four patients with a *BRAF* V600E mutation (Supplementary Fig. 5b, c). In 33% (*n* = 41) of patients, CTFS was reported as <1%, with PAAs found in only 11 of these individuals. A reported PAA was significantly less likely in patients with CTFS < 1% as compared to patients with ≥1% (Fishers exact two-sided test; 27.5% vs 75.4%; *p* < 0.0001). The variant allele frequency (VAF) of alterations demonstrated a bimodal distribution with 442/1642 alterations (27%) having a VAF of <1% and 394/1642 (24%) alterations with a VAF between 45 and 55% (Supplementary Fig. 6A). Only 18 of the alterations with VAF between 45 and 55% were likely oncogenic or oncogenic by OncoKB analysis and the top 10 altered genes with the highest reported VAFs in blood were genes also commonly associated with CHIP (Supplementary Fig. 6B).

**Fig. 5:**
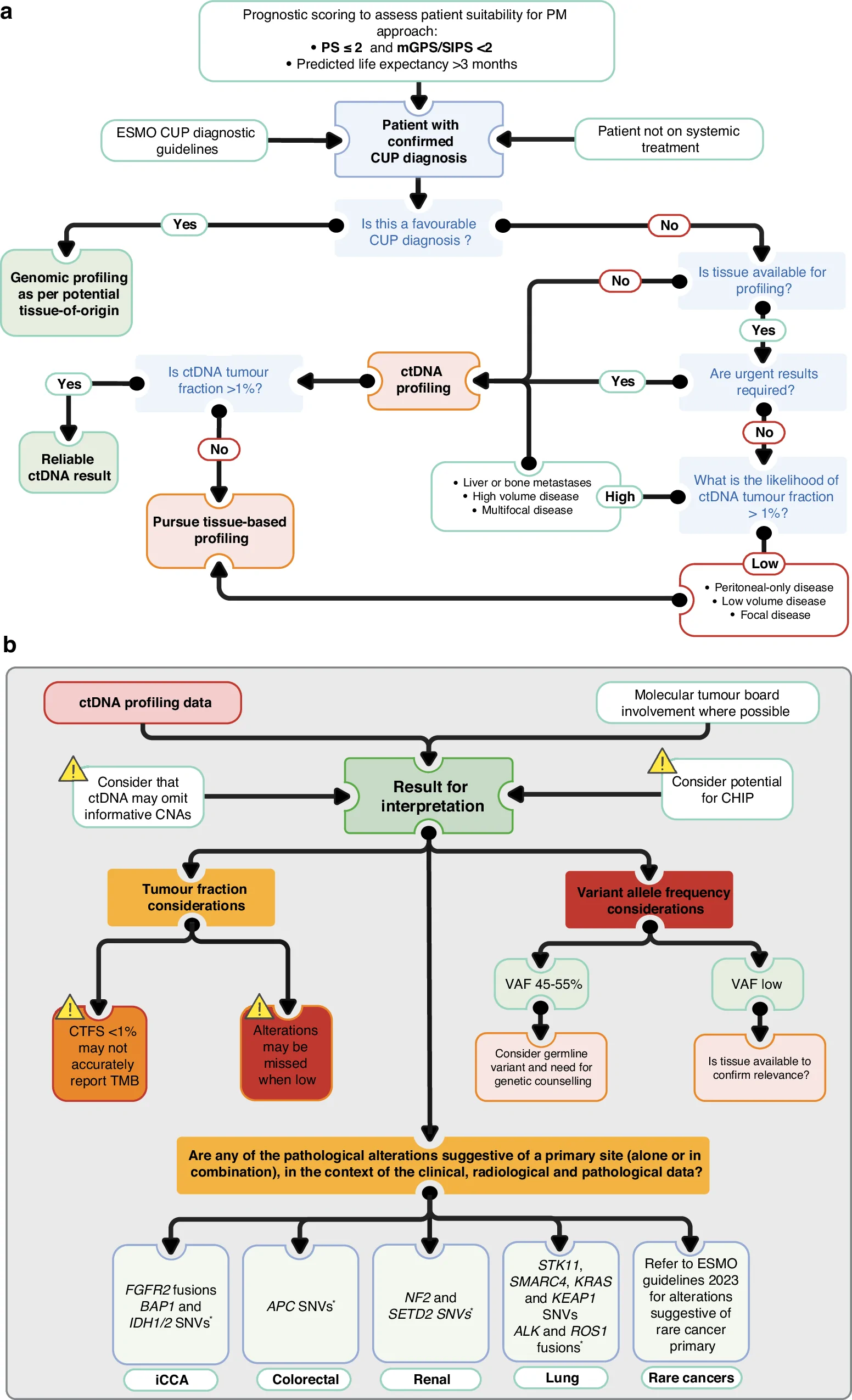
Utilisation and interpretation considerations for molecular profiling in CUP. **a** CUP-COMP decision tree to guide decision-making regarding blood- and tissue-based genomic profiling in patients with a confirmed CUP diagnosis. **b** ctDNA profiling result flow diagram to aid interpretation of genomic profiling data. Note low VAF is dependent on assay, for this study it was considered at <1%. CHIP clonal haematopoiesis of indeterminate potential, CNA copy number alteration, ctDNA circulating tumour DNA, CTFS comprehensive tumour fraction score, CUP cancer of unknown primary, iCCA intrahepatic cholangiocarcinoma, mGPS Modified Glasgow Prognostic Score, PS performance status, SIPS The Scottish Inflammatory Prognostic Score, TMB tumour mutational burden, VAF variant allele frequency. *Commonly reported mutation patterns in CUP literature [5, 29] **see ESMO guidelines [3]. Created in BioRender. Robinson, M. (2026) https://BioRender.com/u86u413.

Finally, to further evaluate clinical utility of blood-based approaches, the CTFS score from baseline blood samples (*n* = 109) was compared to clinical features to assess which patients may benefit most from blood-based approaches (Fig. 5a, b). Patients with liver or bone metastases were more likely to have a CTFS of ≥1% compared to those without (Liver metastases: 36% vs 10%; Pearson’s Chi-squared test; *p* = 0.003; Bone metastases: 38% vs 15%; Pearson’s Chi-squared test; *p* = 0.022) (Supplementary Table 4). CTFS ≥ 1% had no impact on survival (data not shown), and there was no significant difference in the proportion of patients with CTFS ≥ 1% between treatment groups 1–3 (Supplementary Table 3).

## Discussion

CUP-COMP is the first multi-site prospective study in CUP assessing the feasibility and potential clinical utility of a blood-based NGS approach as compared to tissue-based profiling. Here, we present data highlighting the ongoing challenges in attempting tissue-based molecular profiling and demonstrate the clinical utility and feasibility of blood-based profiling in a broad ‘real-world’ population of patients with CUP. Both ‘favourable’ and ‘poor-risk’ CUP subtypes were included, with ECOG PS 0–2, and patients were recruited at any point in their treatment journey. Greater than 50% of patients had PAAs reported, consistent with several retrospective single-institution descriptive studies [15–19]. In our prospective study, one-third of the cohort received a PM approach to treatment, with significant improvement in survival. This supports the growing evidence of successful integration of PM approaches into CUP treatment pathways to aid diagnosis and treatment decisions [20–22]. Most pivotal is the recently published randomised Phase II trial, CUPISCO, demonstrating that a PM approach can improve outcomes in patients diagnosed with ‘poor-risk’ CUP who responded to induction chemotherapy [4, 23]. However, this cohort was restricted to those patients with poor-risk CUP, PS 0–1 and adenocarcinoma. The benefit therefore remains unclear in those patients with poorer performance status, high inflammatory markers, non-adenocarcinoma and ‘favourable’ subtypes of CUP [14].

Although molecular profiling is an increasingly integrated requirement across the advanced cancer spectrum, routine use in CUP is variable internationally, and it is not currently reimbursed or approved in several countries. Where approval is granted, this is mostly for tissue-based NGS or single-gene testing. In England, WGS for CUP was included in the National Genomic Test Directory in the 31st October 2022 publication, however uptake was limited due to several reasons, including requirements for repeated fresh tissue biopsy. Tumour tissue in CUP, with appropriate cellularity and quality for NGS testing, is limited in supply [24]. Therefore, patients often require repeat tumour biopsies to obtain adequate tissue, limiting accessibility to testing as biopsies may be technically unfeasible or unsafe. This study demonstrates the challenges of obtaining adequate tissue for molecular profiling in CUP; with less than 20% of patients having repeat fresh biopsy for WGS attempted and early trial amendments made to facilitate archival tissue molecular profiling. Despite this, there was only a 57% success rate for molecular profiling using archival tissue; comparable to other reported studies where success was reported at 40% in one ‘real-world’ setting [24–27]. Biopsy acquisition is invasive, adds further delays to testing and treating, increases costs and requires extra resources, all of which are factors that likely contributed to the low (18%) rate of repeated biopsy in this study. Blood-based profiling, however, was quick, highly feasible and can overcome the issue of tissue scarcity in a population that has often already had significant delays in the diagnostic process.

This study highlights the value of real-time MTB interpretation in patients with CUP; molecular profiling may not only identify PAAs but may also suggest a potential primary tumour (Fig. 5b) [5, 28, 29]. Indeed, in the CUPISCO dataset, co-occurring mutation patterns clustered patients with molecular features reminiscent of known tumour types despite the strict eligibility criteria of this study [29], and molecular features of a colorectal cancer-like CUP favourable subtype have been defined within ESMO clinical guidelines for some time [8]. In our cohort, 41 patients had a primary tumour diagnosis clinically confirmed or suspected, and in 22% of these cases, molecular profiling contributed to the diagnosis. With the increasing availability of mutation profiling from large cohorts of patients with CUP and known tumours, there is a clinical need for guidelines to guide the interpretation of co-mutation patterns to aid diagnosis [5].

Just over a quarter of the CUP-COMP cohort went on to have a PM treatment, either through the determination of a primary tumour, entry into a clinical trial or compassionate access. However only 16/117 (14%) patients received treatments that directly targeted a PAA, which is less than reported in the molecularly guided arm of CUPISCO (26%) but similar to larger pan-cancer PM trials [30, 31]. It is predicted that the proportion of patients that will receive targeted therapies is expected to increase as more alterations become actionable as targeted drug development evolves over time. Of note, 37% of the CUP-COMP cohort had no SACT (BSC), with a higher proportion of patients with ECOG PS 2 in this group, and median survival of only 4.5 months (3.7 months if treatment naïve). This highlights the need for appropriate consideration of genomic testing and reserving testing for patients who are most likely to benefit from treatment. A PM approach was associated with an improved OS in this all-comers real-world data. Survival comparison data suggested that the treatment naïve group are more likely to benefit from this approach and upfront molecular profiling can support a primary tumour diagnosis, which will directly impact first-line treatment.

Retrospective comparative analysis and evaluation of oncogenicity across blood-based profiling has identified key considerations for interpretation when using blood-based profiling for clinical decision-making in CUP (Fig. 5b). Blood-based profiling can miss potentially informative CNAs that are present in tissue and detect potential actionable SNVs at low VAF in genes commonly associated with CHIP or germline mutations. These findings can result in falsely identification of a PAA attributable to the tumour and emphasises the need for an experienced MTB used to interpreting blood-based results. A further study is planned to include confirmatory germline analysis across the cohort to further characterise both potential CHIP and germline frequency of alterations. Our data showed that CTFS of <1% (present in 36% of cohort) impacts the likelihood of a PAA being reported and under-reporting of high-TMB. Therefore, the most appropriate molecular profiling for an individual should be considered before requesting (Fig. 5a). In our cohort, the presence of liver metastases or bone metastases significantly impacted the likelihood of a CTFS ≥ 1%. We know that burden and sites of disease can impact ctDNA content as well as tumour type-dependent factors [32], emphasising the importance of MTB input in relation to ctDNA results. In England, recent approvals of ctDNA testing in Non-small cell lung cancer and breast cancer [33] highlight the growing need to interpret blood-based results in the context of tumour fraction as such testing becomes more common-place.

Real-time MTB discussions considered broad actionability criteria, including early-phase clinical trial entry, resulting in a much higher actionability than reported in other studies (63%) [4, 29, 34]. Although no formal actionability criteria or algorithm was used in the MTB, retrospective application of OncoKB levels 1-3b criteria to the blood-only molecular profiling cohort resulted in similar proportion of patients with PAAs as found in real-time MTB discussions (58%); 19% of patients harboured PAAs with level 1 evidence of actionability (tumour-agnostic indication). However, 15% of the level 1 evidence is applying a bTMB cut-off the same as tissue (≥10 Muts/Mb). The predictive ability of bTMB-high for immunotherapy response varies across different tumour types and immunotherapies, and tests vary in their calculation. There is debate as to the appropriate cut-off to employ for ctDNA, especially in the context of low tumour fraction [35]. In the CUPISCO trial, more than two-thirds of the patients in the MGT group (without an actionable alteration) received immunotherapy with chemotherapy. In our study more than two-thirds of the patients in the PM group received immunotherapy either within or outside of clinical trials. Despite the debate over reliability of predictive immunotherapy biomarkers, this signal calls for properly designed clinical trials to explore the efficacy of immunotherapy in this group of patients.

Four patients harboured a *BRAF* V600E mutation which was recently deemed targetable with BRAF inhibitors in the tumour-agnostic setting [36]. Interestingly of these 4 patients, 1 had a suspected melanoma following molecular profiling and another had a suspected GI malignancy. The two remaining patients had no primary tumour suspected or confirmed. Despite tumour agnostic approvals for BRAF inhibitors in this setting, it is known that the context of the tumour origins plays a pivotal role in selecting combination treatment and predicting response rates [36]. The remaining patients that harboured PAAs constituted level 3a or 3b evidence of therapeutic actionability. Most cases were level 3b – the biomarker is predictive of response but in a different tumour type indication. Without a primary tumour diagnosis, the true actionability of the biomarker remains uncertain. For some patients, the presence of such a biomarker is highly indicative of the primary site. For example, 3 patients harboured *FGFR2* fusions and subsequently had a confirmed or highly suspected diagnosis of intrahepatic cholangiocarcinoma (iCCA). FGFR inhibitors are SoC and approved in the UK and USA for iCCA, and therefore the molecular pattern and primary diagnosis changed SoC therapeutic options significantly [37, 38]. Another patient with suspected breast cancer harboured both *ERBB2* amplification and *PIK3CA* mutation, both level 1 evidence in breast cancer for response to HER2 antagonists and alpelisib, respectively [39, 40].

There were several limitations to this study. This study was not randomised, so post-hoc treatment groupings were exploratory in nature and not matched for clinical characteristics such as ECOG PS or prognostic biomarkers, and therefore potential bias may impact results. Although ECOG PS was significantly worse in the BSC group, SIPS prognostic scores were not different between treatment groups, supporting there may be independent benefit on survival of the PM approach compared to chemotherapy. However, it remains that there are several other confounders in addition to the ECOG PS bias that may have impacted the survival analysis including, extent and sites of disease and treatment selection bias. Post-hoc statistical survival comparisons between treatment groups were underpowered. In addition, tissue profiling was not routinely performed at baseline, although at the outset, repeat tissue biopsy for WGS was mandated. This meant that a comparison of the real-time clinical utility of blood and tissue, particularly WGS, including turnaround time and success of profiling, was not feasible. An amendment was made early in the trial to include FFPE profiling if blood-based testing failed, as it was apparent that timely repeat biopsies were limiting recruitment and were difficult to obtain. This has confounded attempts to compare molecular concordance between modalities and in those patients that did undergo both FM blood and tissue profiling, retrospective comparative analysis has been shown, however we acknowledge the time between tissue biopsy and time of blood draw was highly variable, with some patients receiving therapy and therefore the effect of tumour evolution affects direct comparisons. The challenges experienced in successfully profiling tissue in this study do however support the clinical utility of offering blood-based profiling over tissue in some patients with CUP. Future retrospective work is planned to evaluate the potential added value of WGS over panel testing, including inferring tissue-of-origin through genome-wide approaches.

We recognise that this cohort of patients with CUP represents a selective and heterogeneous group of patients, reflected in the relatively good 12-month median OS of 9.5 months. A significant proportion of patients could be categorised as favourable prognosis group (single site, lymph node limited, primary found/highly suspected) likely due to more permissive eligibility criteria but representing a more “real world” cohort. Given the disparity in organisation and provision of CUP services globally we demonstrate the feasibility of incorporating NGS early in the CUP pathway ensuring timely stratification of patients to potential PM approaches.

Due to the nature of the study, it is likely and appropriate that only those patients with better prognostic features were selected for the study, and survival is broadly similar to other reported studies evaluating PM approaches in CUP cohorts. Recently reported outcomes from an Australian study demonstrated a site-specific approach improved OS compared to treatment with chemotherapy alone (Median OS 20.2 vs 10.9 months, respectively) [22]. CUPISCO interim OS data has demonstrated a median OS of 14.7 months for chemotherapy responders and 14.0 months in non-responders for the PM group compared to 11.0 months for responders and 5.2 for non-responders, in those patients who continued chemotherapy alone [4].

## Conclusions

In conclusion, this study highlights the feasibility and clinical utility of liquid biopsies in patients diagnosed with CUP. In a disease type with limited treatment options, implementing a PM approach can dramatically alter potential therapeutic choices either through changing primary tumour diagnosis and/or highlighting potential PM options. In this cohort, blood testing was highly successful, but there remains a proportion of patients where tumour fraction was low, risking inaccurate results and for whom tissue profiling would be the preferred option, if available. Where tissue is lacking, or where there may be delays in accessing tissue, opting for blood-based profiling is recommended (see Fig. 5a, b).

## Supplementary information

Supplementary Material

Supplementary Tables

CUP-COMP trial protocol

## Data Availability

All the data generated or analysed during this study are included in this published article and supplementary files or are available from the corresponding author upon reasonable request. Genome data from tissue and blood have been deposited at the European Genome-phenome Archive (EGA) which is hosted at the EBI and the CRG under controlled access (Study ID accession number: EGAS50000001821). Data access for non-commercial purposes may be submitted through the EGA Data Access Committee (DAC accession: EGAC50000000610) or by contacting the DAC administrator (matthew.concannon@nhs.net), The Christie NHS Foundation Trust.

## References

[1] Binder C, Matthes KL, Korol D, Rohrmann S, Moch H. Cancer of unknown primary-Epidemiological trends and relevance of comprehensive genomic profiling. Cancer Med. 2018;7:4814–24. 10.1002/cam4.1689 PMC6144156 30019510

[2] CRUK Cancer Research UK. Cancer of Unknown Primary Cancer Statistics [Internet]. Updated 2020 [Available from: https://www.cancerresearchuk.org/health-professional/cancer-statistics/statistics-by-cancer-type/cancer-of-unknown-primary.

[3] Krämer A, Bochtler T, Pauli C, Baciarello G, Delorme S, Hemminki K, et al. Cancer of unknown primary: ESMO Clinical Practice Guideline for diagnosis, treatment and follow-up. Ann Oncol. 2022;34:228–46. 10.1016/j.annonc.2022.11.013 36563965

[4] Krämer A, Bochtler T, Pauli C, Shiu K-K, Cook N, de Menezes JJ, et al. Molecularly guided therapy versus chemotherapy after disease control in unfavourable cancer of unknown primary (CUPISCO): an open-label, randomised, phase 2 study. Lancet. 2024;404:527–39. 10.1016/S0140-6736(24)00814-6 39096924

[5] Posner A, Prall OW, Sivakumaran T, Etemadamoghadam D, Thio N, Pattison A, et al. A comparison of DNA sequencing and gene expression profiling to assist tissue of origin diagnosis in cancer of unknown primary. J Pathol. 2022;259:81–92. 10.1002/path.6022 PMC10099529 36287571

[6] Liu X, Zhang X, Jiang S, Mo M, Wang Q, Wang Y, et al. Site-specific therapy guided by a 90-gene expression assay versus empirical chemotherapy in patients with cancer of unknown primary (Fudan CUP-001): a randomised controlled trial. Lancet Oncol. 2024;25:1092–102. 10.1016/S1470-2045(24)00313-9 39068945

[7] Hainsworth JD, Fizazi K. Treatment for patients with unknown primary cancer and favorable prognostic factors. Semin Oncol. 2009;36:44–51. 10.1053/j.seminoncol.2008.10.006 19179187

[8] Fizazi K, Greco FA, Pavlidis N, Daugaard G, Oien K, Pentheroudakis G. Cancers of unknown primary site: ESMO Clinical Practice Guidelines for diagnosis, treatment and follow-up. Ann Oncol. 2015;26:v133–v8. 10.1093/annonc/mdr389 21908507

[9] Horak P, Griffith M, Danos AM, Pitel BA, Madhavan S, Liu X, et al. Standards for the classification of pathogenicity of somatic variants in cancer (oncogenicity): Joint recommendations of Clinical Genome Resource (ClinGen), Cancer Genomics Consortium (CGC), and Variant Interpretation for Cancer Consortium (VICC). Genet Med. 2022;24:986–98. 10.1016/j.gim.2022.01.001 PMC9081216 35101336

[10] Garrett A, Callaway A, Durkie M, Cubuk C, Alikian M, Burghel GJ, et al. Cancer variant interpretation group UK (CanVIG-UK): an exemplar national subspecialty multidisciplinary network. J Med Genet. 2020;57:829–34. 10.1136/jmedgenet-2019-106759 PMC7691806 32170000

[11] Li MM, Datto M, Duncavage EJ, Kulkarni S, Lindeman NI, Roy S, et al. Standards and Guidelines for the Interpretation and reporting of sequence variants in cancer: a joint consensus recommendation of the Association for Molecular Pathology, American Society of Clinical Oncology, and College of American pathologists. J Mol Diagn. 2017;19:4–23. 10.1016/j.jmoldx.2016.10.002 PMC5707196 27993330

[12] Richards S, Aziz N, Bale S, Bick D, Das S, Gastier-Foster J, et al. Standards and guidelines for the interpretation of sequence variants: a joint consensus recommendation of the American College of Medical Genetics and Genomics and the Association for Molecular Pathology. Genet Med. 2015;17:405–24. 10.1038/gim.2015.30 PMC4544753 25741868

[13] Kuzbari Z, Bandlamudi C, Loveday C, Garrett A, Mehine M, George A, et al. Germline-focused analysis of tumour-detected variants in 49,264 cancer patients: ESMO Precision Medicine Working Group recommendations. Ann Oncol. 2023;34:215–27. 10.1016/j.annonc.2022.12.003 36529447

[14] Harvey S, Stares M, Scott JA, Thottiyil TJV, Conway AM, Haigh R, et al. Biomarkers of systemic inflammation provide additional prognostic stratification in cancers of unknown primary. Cancer Med. 2024;13:e6988. 10.1002/cam4.6988 PMC10895198 38404120

[15] Gatalica Z, Millis SZ, Vranic S, Bender R, Basu GD, Voss A, et al. Comprehensive tumor profiling identifies numerous biomarkers of drug response in cancers of unknown primary site: analysis of 1806 cases. Oncotarget. 2014;5:12440–7. 10.18632/oncotarget.2574 PMC4322997 25415047

[16] Gatalica Z, Xiu J, Swensen J, Vranic S. Comprehensive analysis of cancers of unknown primary for the biomarkers of response to immune checkpoint blockade therapy. Eur J Cancer. 2018;94:179–86. 10.1016/j.ejca.2018.02.021 29571084

[17] Löffler H, Pfarr N, Kriegsmann M, Endris V, Hielscher T, Lohneis P, et al. Molecular driver alterations and their clinical relevance in cancer of unknown primary site. Oncotarget. 2016;7:44322–9. 10.18632/oncotarget.10035 PMC5190099 27322425

[18] Varghese AM, Arora A, Capanu M, Camacho N, Won HH, Zehir A, et al. Clinical and molecular characterization of patients with cancer of unknown primary in the modern era. Ann Oncol. 2017;28:3015–21. 10.1093/annonc/mdx545 PMC5834064 29045506

[19] Kato S, Krishnamurthy N, Banks KC, De P, Williams K, Williams C, et al. Utility of genomic analysis in circulating tumor DNA from patients with carcinoma of unknown primary. Cancer Res. 2017;77:4238–46. 10.1158/0008-5472.CAN-17-0628 PMC5729906 28642281

[20] Weiss L, Heinrich K, Zhang D, Dorman K, Rühlmann K, Hasselmann K, et al. Cancer of unknown primary (CUP) through the lens of precision oncology: a single institution perspective. J Cancer Res Clin Oncol. 2023;149:8225–34. 10.1007/s00432-023-04741-y PMC10374717 37062035

[21] Jacquin N, Kamal M, Bieche I, Dupain C, Guillou I, Rouleau E, et al. Effect of national multidisciplinary tumor boards on diagnostic stratification and therapeutic management in cancers of unknown primary. J Clin Oncol. 2023;41:e13666–e.

[22] van Mourik A, Tonkin-Hill G, O’Farrell J, Waller S, Tan L, Tothill RW, et al. Six-year experience of Australia’s first dedicated cancer of unknown primary clinic. Br J Cancer. 2023;129:301–8. 10.1038/s41416-023-02254-6 PMC10338450 37225894

[23] Bochtler T, Kramer A, Pauli C, Durán-Pacheco G, Arslan C, Bigot F, et al. Outcomes of patients (pts) with unfavourable, non-squamous cancer of unknown primary (CUP) progressing after induction chemotherapy (CTX) in the global, open-label, phase II CUPISCO study. Ann Oncol. 2024;35:S238–S308.

[24] Huey RW, Shah AT, Reddi HV, Dasari P, Topham JT, Hwang H, et al. Feasibility and value of genomic-profiling in cancer of unknown primary: real-world evidence from prospective profiling study. J Natl Cancer Inst. 2023;115:994–97. 10.1093/jnci/djad095 PMC10407690 37202363

[25] Conway AM, Morris GC, Smith S, Manoharan P, Mitchell C, Backen A, et al. editors. Intrahepatic cholangiocarcinoma (iCCA) hidden amongst the unknown: a retrospective analysis of cancer of unknown primary (CUP) cases from a tertiary cancer centre. Ann Oncol. 2021;32:S380–1.

[26] Pauli C, Bochtler T, Mileshkin L, Baciarello G, Losa F, Ross JS, et al. A challenging task: identifying patients with cancer of unknown primary (CUP) according to ESMO Guidelines: the CUPISCO trial experience. Oncologist. 2021;26:e769–e79. 10.1002/onco.13744 PMC8100559 33687747

[27] Zaun G, Borchert S, Metzenmacher M, Lueong S, Wiesweg M, Zaun Y, et al. Comprehensive biomarker diagnostics of unfavorable cancer of unknown primary to identify patients eligible for precision medical therapies. Eur J Cancer. 2024;200:113540. 10.1016/j.ejca.2024.113540 38316065

[28] Benvenuti S, Milan M, Geuna E, Pisacane A, Senetta R, Gambardella G, et al. Cancer of unknown primary: genetic evidence for a novel nosological entity? A case report. EMBO Mol Med. 2020;12:EMMM201911756. 10.15252/emmm.201911756 PMC7338804 32511869

[29] Westphalen CB, Federer-Gsponer J, Pauli C, Karapetyan AR, Chalabi N, Durán-Pacheco G, et al. Baseline mutational profiles of patients with carcinoma of unknown primary origin enrolled in the CUPISCO study. ESMO Open. 2023;8:102035. 10.1016/j.esmoop.2023.102035 PMC10774891 37922692

[30] Rothwell DG, Ayub M, Cook N, Thistlethwaite F, Carter L, Dean E, et al. Utility of ctDNA to support patient selection for early phase clinical trials: the TARGET study. Nat Med. 2019;25:738–43. 10.1038/s41591-019-0380-z 31011204

[31] Middleton G, Robbins H, Andre F, Swanton C. A state-of-the-art review of stratified medicine in cancer: towards a future precision medicine strategy in cancer. Ann Oncol. 2022;33:143–57. 10.1016/j.annonc.2021.11.004 34808340

[32] Bettegowda C, Sausen M, Leary RJ, Kinde I, Wang Y, Agrawal N, et al. Detection of circulating tumor DNA in early- and late-stage human malignancies. Sci Transl Med. 2014;6:224ra24–ra24. 10.1126/scitranslmed.3007094 PMC4017867 24553385

[33] Wise J. Liquid biopsy for lung and breast cancers becomes available across the NHS in England. BMJ. 2025;389:r1130. 10.1136/bmj.r1130 40456592

[34] Ross JS, Sokol ES, Moch H, Mileshkin L, Baciarello G, Losa F, et al. Comprehensive genomic profiling of carcinoma of unknown primary origin: retrospective molecular classification considering the CUPISCO study design. Oncologist. 2021;26:e394–402. 10.1002/onco.13597 PMC7930409 33219618

[35] Tsui DWY, Cheng ML, Shady M, Yang JL, Stephens D, Won H, et al. Tumor fraction-guided cell-free DNA profiling in metastatic solid tumor patients. Genome Med. 2021;13:96. 10.1186/s13073-021-00898-8 PMC8165771 34059130

[36] Hyman DM, Puzanov I, Subbiah V, Faris JE, Chau I, Blay JY, et al. Vemurafenib in multiple nonmelanoma cancers with BRAF V600 mutations. N Engl J Med. 2015;373:726–36. 10.1056/NEJMoa1502309 PMC4971773 26287849

[37] Abou-Alfa GK, Sahai V, Hollebecque A, Vaccaro G, Melisi D, Al-Rajabi R, et al. Pemigatinib for previously treated, locally advanced or metastatic cholangiocarcinoma: a multicentre, open-label, phase 2 study. Lancet Oncol. 2020;21:671–84. 10.1016/S1470-2045(20)30109-1 PMC8461541 32203698

[38] Javle M, Lowery M, Shroff RT, Weiss KH, Springfeld C, Borad MJ, et al. Phase II study of BGJ398 in patients with FGFR-Altered advanced cholangiocarcinoma. J Clin Oncol. 2018;36:276–82. 10.1200/JCO.2017.75.5009 PMC6075847 29182496

[39] Jallah JK, Dweh TJ, Anjankar A, Palma O. A review of the advancements in targeted therapies for breast cancer. Cureus. 2023;15:e47847. 10.7759/cureus.47847 PMC10679843 38022130

[40] Chakravarty D, Gao J, Phillips SM, Kundra R, Zhang H, Wang J, et al. OncoKB: a precision oncology knowledge base. JCO Precis Oncol. 2017;1:1–16. 10.1200/PO.17.00011 PMC5586540 28890946

