# Supplementary material for: A prospective national precision medicine trial evaluating the feasibility of blood-based molecular profiling in patients with Cancer of Unknown Primary (CUP-COMP)
Source: Br J Cancer. 2026 Jul 20;135(8):1255–66. doi: 10.1038/s41416-026-03519-6 (PMC13642309; doi:10.1038/s41416-026-03519-6)
Supplement: Supplementary file 1 [file 41416_2026_3519_MOESM1_ESM.pdf]

SUPPLEMENTARY MATERIAL for:

A prospective national precision medicine trial comparing blood and tissue profiling in patients with Cancer of Unknown Primary (CUPCOMP)

Corresponding author: Natalie.cook17@nhs.net

Supplementary Figures 1-6, Supplementary Methods, Supplementary References

Supplementary Figure 1: Confirmed and suspected primary tumour diagnoses made during study, colour coded by confirmed diagnosis or suspected and whether molecular or clinical data led to primary tumour suspicion. Note: 3 with iCCA confirmed diagnosis were further supported by molecular data. *Other=Adenocarcinoma somatic transformation of previous germ cell tumour. GI=gastrointestinal; iCCA=intrahepatic cholangiocarcinoma; RCC=Renal Cell Carcinoma; SCC=Squamous Cell Carcinoma. **Supplementary Figure 2:** Survival analysis within Precision Medicine Group 3. Excluding 3 patients that received immunotherapy not directed to change in diagnosis nor biomarker, patients were split by those receiving treatment based on change in diagnosis (n=16, Group A, site-directed, blue) or suspected diagnosis or those receiving treatment directed to a detected potentially actionable alteration (PAA) (n=12, Group B, PAA-directed, red). Of note 4 patients within Group A had both a change of diagnosis and a treatment directed to a PAA. CI=Confidence Intervals; HR=Hazard Ratio. **Supplementary Figure 3:** Survival analysis by treatment group. (A) In the treatment naïve cohort (n=75). (B) In the pre-treated cohort (n=35). BSC=Best Supportive Care; CI=Confidence Intervals; Doublet CT=Doublet chemotherapy; HR=hazard Ratio; Precision Medicine (PM). **Supplementary Figure 4: Comparison of alterations in tissue and blood (n=51). (A)** Proportional Venn diagrams of all actionable alterations found in patients where both tissue and blood FM panel testing was performed (n=51). Venn restricted by alterations oncogenicity and actionability as determined by OncoKB. Blue=found in tissue only; Red=found in blood only; crossover=found in both modalities. **(B)** Individual patient level concordance rate of alterations found in blood and tissue (n=51) against comprehensive tumour fraction score (CTFS); Pearsons's correlation R2=0.1139, *p*-value=0.0154. **Supplementary Figure 5:** Retrospective reporting of potentially actionable alterations as determined by OncoKB from blood-based profiling across the cohort (n=112). **(A)** Potentially actionable alterations per patient colour-coded by alteration type. **(B)** Potentially actionable alterations by gene colour-coded by alteration type. **(C)** Proportion of CUP cohort with successful blood molecular profiling (n=112) with potentially actionable alterations and highest level of actionability for alterations as per OncoKB levels of therapeutic

actionability.(40) Outer pie – broad level of evidence; inner pie – detail of actionability. Left- OncoKB actionability scale. *2 patients with BRAF V600E and high-TMB also had MSI-high. b-TMB=blood Tumour Mutation Burden; CNA=Copy Number Alterations; FDA=Food and Drug Administration; MSI=Microsatellite Instability; SNV=Single Nucleotide Variants; SV=Structural Variants. **Supplementary Figure 6: (A)** Frequency of alterations (SNVs and structural variants SVs) found in blood across entire cohort (n=112) by variant allele frequency (VAF). Colour coded by oncogenicity. **(B)** Top 10 altered genes by highest Variant Allele Frequency (VAF) sorted by highest median VAF, number at top = number of alterations. **Supplementary Methods. Supplementary References.**

Supplementary Tables 1-4.xlxs: Online supplementary tables 1-4 Supplementary Table 1: Molecular profiling metrics from blood and tissue (n=117). **Supplementary Table 2:** Suspected diagnosis, treatment groups and treatment outcomes per patient. **Supplementary Table 3:** Baseline characteristics by treatment group. **Supplementary Table 4:** Baseline characteristics by Comprehensive Tumour Fraction Score (CTFS).

SUPPLEMENTARY FIGURES

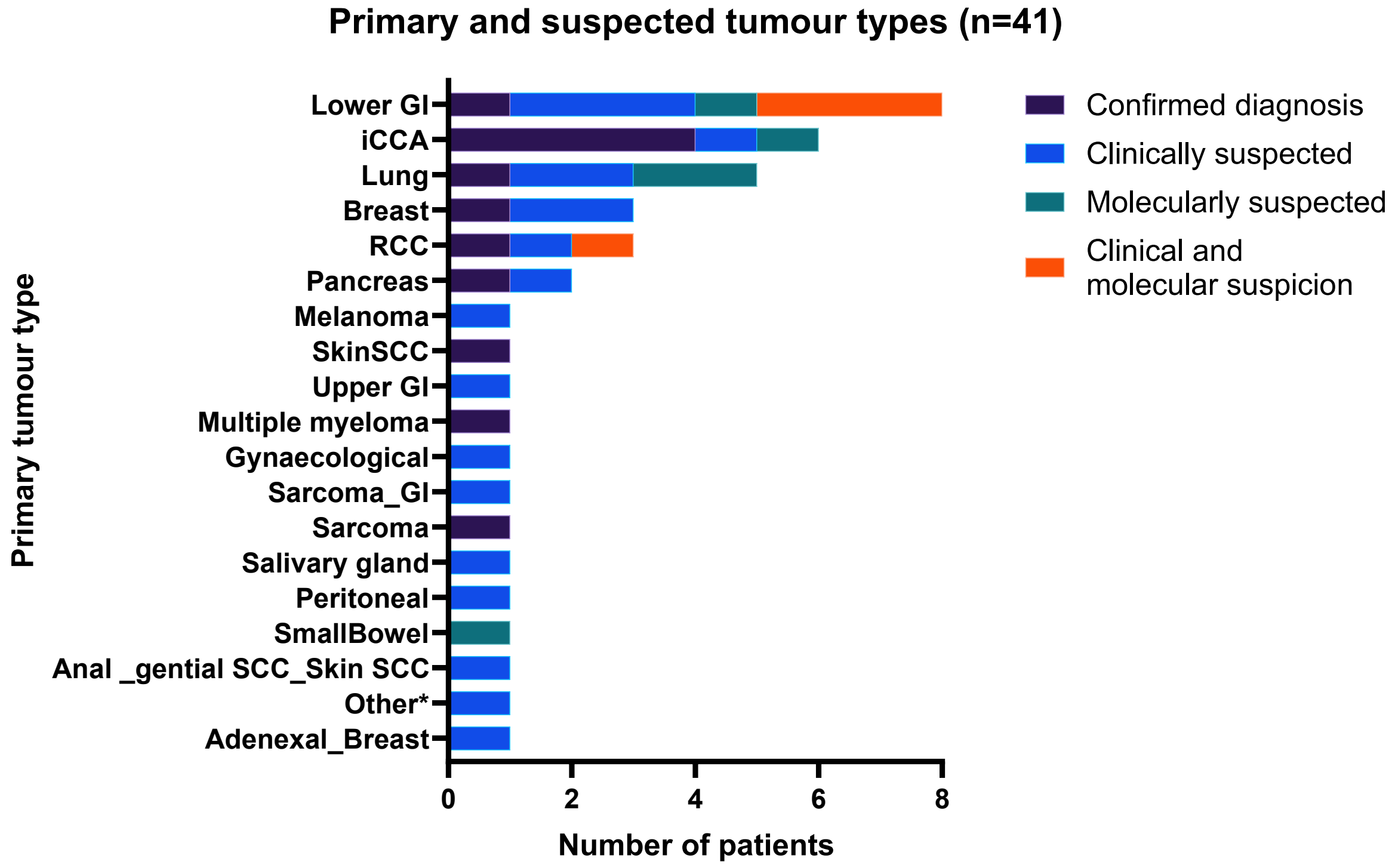

Supplementary Figure 1: Confirmed and suspected primary tumour diagnoses made during study, colour coded by confirmed diagnosis or suspected and whether molecular or clinical data led to primary tumour suspicion. Note: 3 patients with confirmed diagnosis of iCCA were further supported by molecular data. 1 patient with molecularly suspected small bowel cancer had peritoneal primary as clinical suspicion. *Other=Adenocarcinoma somatic transformation of previous germ cell tumour. GI=gastrointestinal; iCCA=intrahepatic cholangiocarcinoma; RCC=Renal Cell Carcinoma; SCC=Squamous Cell Carcinoma

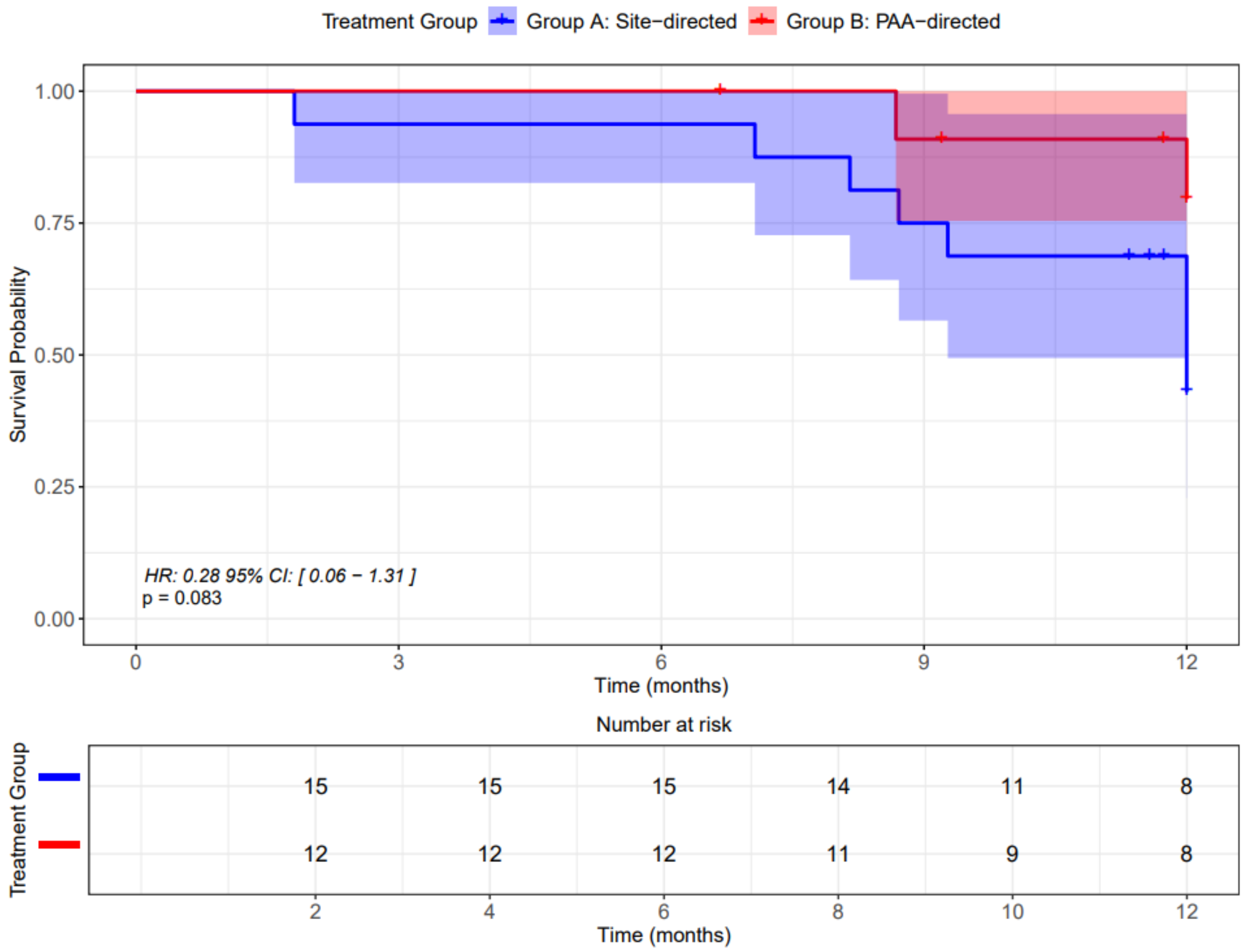

Supplementary Figure 2: Survival analysis within Precision Medicine Group 3. Excluding 3 patients that received immunotherapy not directed to change in diagnosis nor biomarker, patients were split by those receiving treatment based on change in diagnosis (n=16, Group A, site-directed, blue) or suspected diagnosis or those receiving treatment directed to a detected potentially actionable alteration (PAA) (n=12, Group B, PAA-directed, red). Of note 4 patients within Group A had both a change of diagnosis and a treatment directed to a PAA. CI=Confidence Intervals; HR=Hazard Ratio.

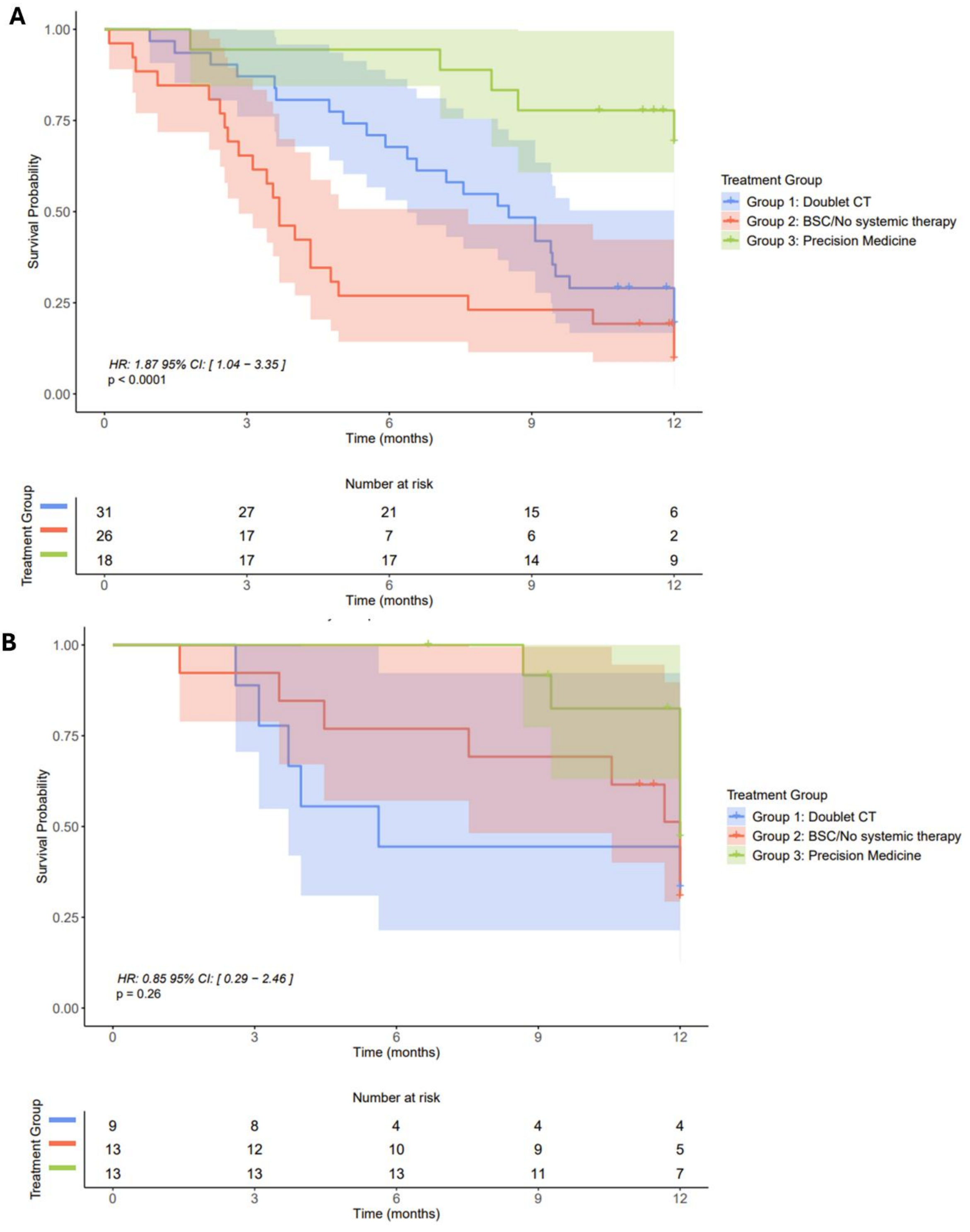

Supplementary Figure 3: Survival analysis by treatment group. (A) In the treatment naïve cohort (n=75). (B) In the pre-treated cohort (n=35). HR=hazard Ratio; CI=Confidence Intervals; Doublet CT=Doublet chemotherapy; BSC=Best Supportive Care; Precision Medicine (PM).

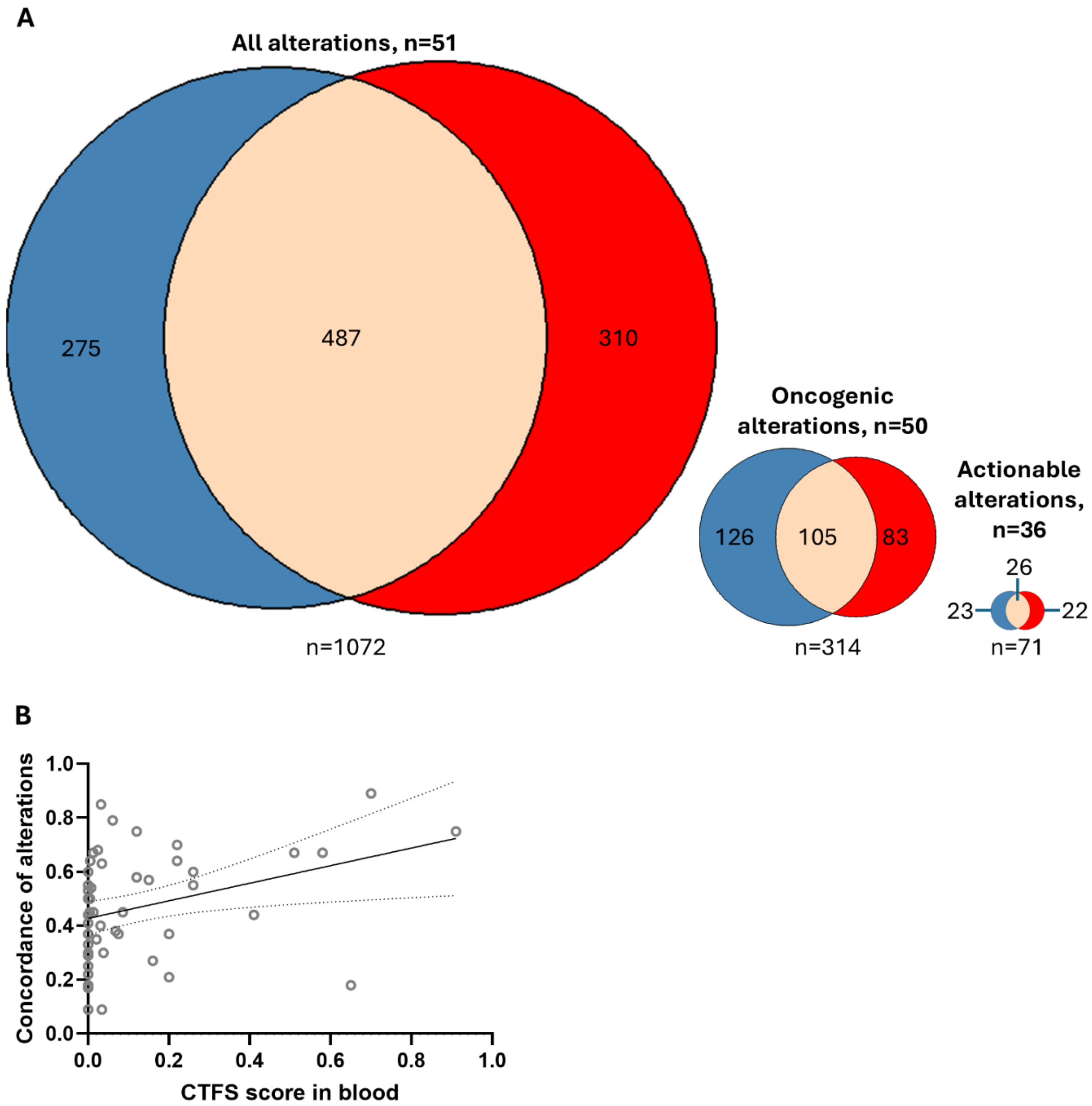

Supplementary Figure 4: Comparison of alterations in tissue and blood (n=51). (A) Proportional Venn diagrams of all actionable alterations found in patients where both tissue and blood FM panel testing was performed (n=51). Venn restricted by alterations oncogenicity and actionability as determined by OncoKB. Blue=found in tissue only; Red=found in blood only; crossover=found in both modalities. **(B)** Individual patient level concordance rate of alterations found in blood and tissue (n=51) against comprehensive tumour fraction score (CTFS); Pearsons's correlation R2=0.1139, *p*-value=0.0154.

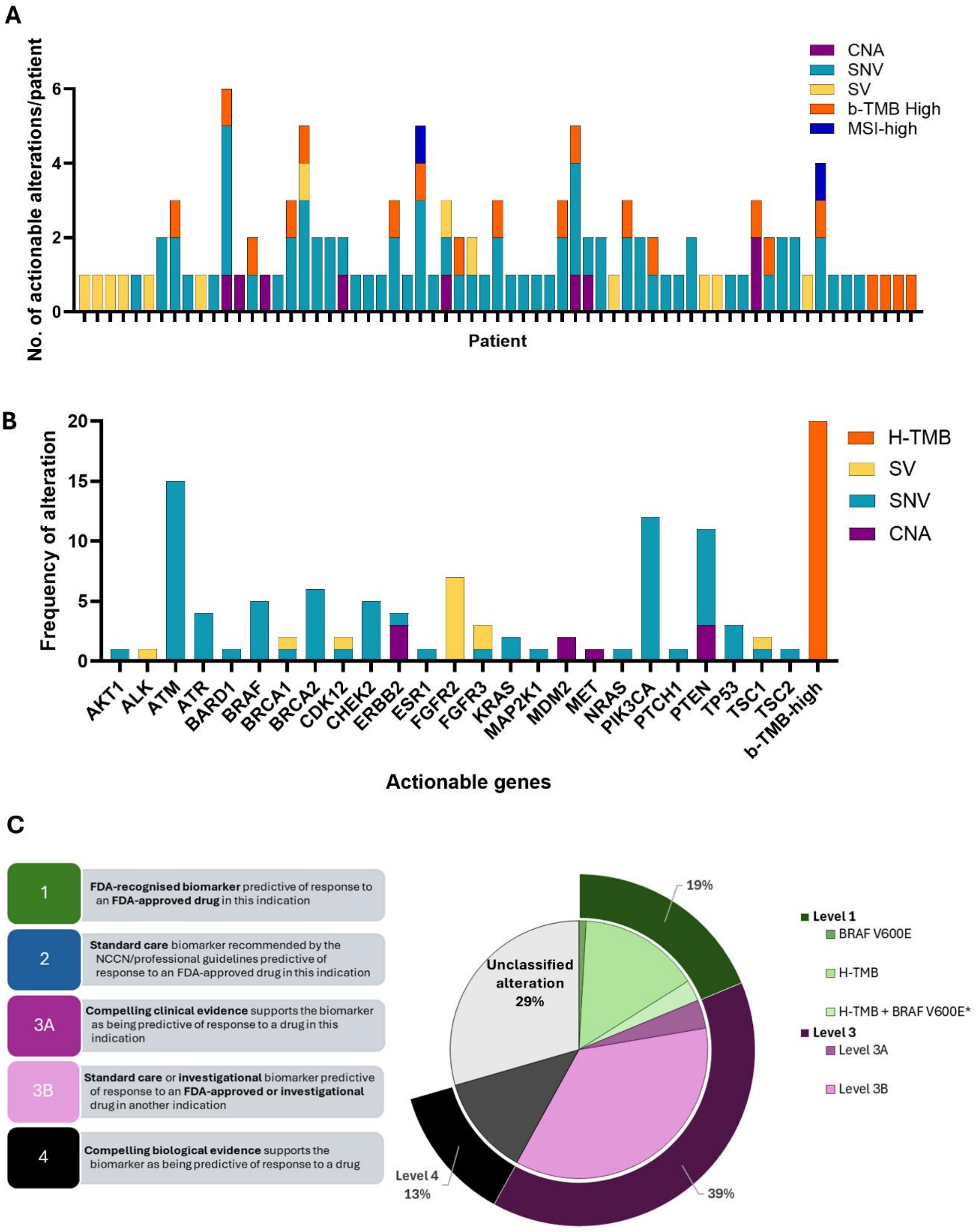

Supplementary Figure 5: Retrospective reporting of potentially actionable alterations as determined by OncoKB from blood-based profiling across the cohort (n=112). **(A)** Potentially actionable alterations per patient colour-coded by alteration type. **(B)** Potentially actionable alterations by gene colour-coded by alteration type. **(C)** Proportion of CUP cohort with successful blood molecular profiling (n=112) with potentially actionable alterations and highest level of actionability for alterations as per OncoKB levels of therapeutic actionability.[1] Outer pie – broad level of evidence; inner pie – detail of actionability. Left- OncoKB actionability scale. *2 patients with BRAF V600E and high-TMB also had MSI-high. CNA=Copy Number Alterations; SNV=Single Nucleotide Variants; SV=Structural Variants; b-TMB=blood Tumour Mutation Burden; MSI=Microsatellite Instability; FDA=Food and Drug Administration.

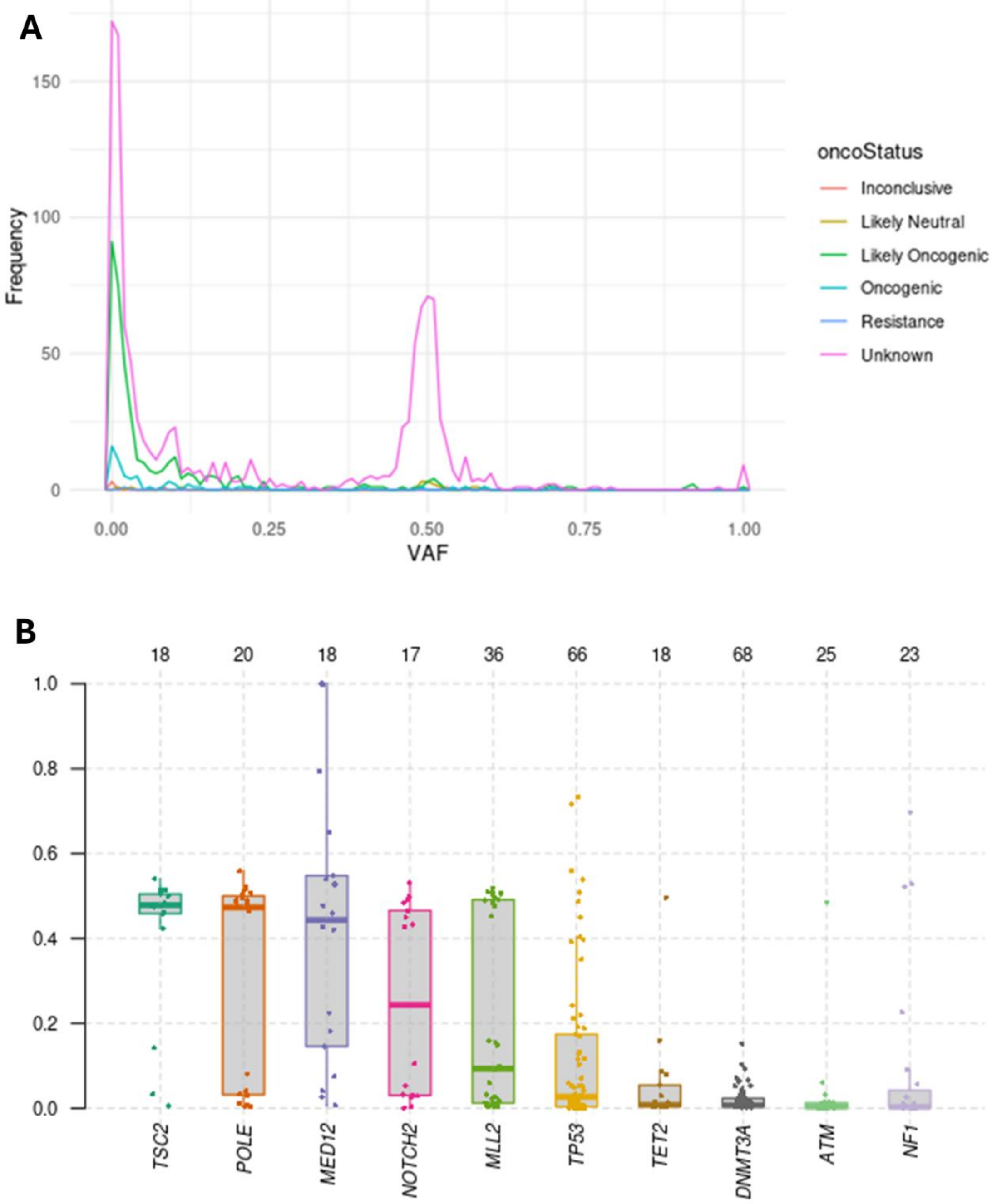

Supplementary Figure 6: (A) Frequency of alterations (SNVs and structural variants SVs) found in blood across entire cohort (n=112) by variant allele frequency (VAF). Colour coded by oncogenicity. **(B)** Top 10 altered genes by highest Variant Allele Frequency (VAF) sorted by highest median VAF, number at top = number of alterations.

SUPPLEMENTARY METHODS

Recruiting sites

Trial recruitment occurred across seven UK sites with good geographical spread. Sites included:

- The Christie NHS Foundation Trust, Manchester;
- Velindre Cancer Centre, Velindre University NHS Trust, Cardiff;
- Torbay and South Devon NHS Foundation Trust, Torquay;
- Royal United Hospitals Bath NHS Foundation Trust, Bath;
- University College London NHS Foundation Trust, London;
- Edinburgh Cancer Centre, Western General Hospital, Edinburgh;
- The Clatterbridge Cancer Centre NHS Foundation Trust, Liverpool.

Primary and Secondary Outcome measures

Primary outcomes measures:

1. To sequence tumour tissue and circulating tumour DNA from approximately 100- 140 patients with CUP in order to evaluate the activation state of various oncogenic pathways and improve treatment stratification approaches;
2. To establish a genomic reporting mechanism whereby clinically relevant and potentially 'actionable' abnormalities found during sequencing/molecular characterisation can be reported to patients.

Secondary Outcomes Measures:

1. To obtain archival tumour specimens, fresh tissue and sequential blood samples from approximately 100 – 140 patients with CUP
2. The comparative assessment of alternative assay methods (such as ctDNA) in parallel with whole genome sequencing in the diagnosis and treatment stratification for patients with CUP (see trial protocol for further details).

Optional Consent

Patients completed an optional consent form for genetic result disclosure regarding: a) results that may have significance for biological family members and/or b) results that are not related to cancer but may have potential medical impact. Initial inclusion criteria mandated acquisition of an additional fresh tissue biopsy for Whole Genome Sequencing (WGS). However, this was amended to an optional consent as it was identified to be a barrier to recruitment. In addition,

patients could optionally consent for their samples to be used in future research and/or to grow cancer cells in the laboratory for animal experimentation.

Foundation Medicine Next Generation Sequencing Testing in blood and tissue

Tissue based genomic profiling was undertaken FFPE tissue using the FoundationOne®CDx assay (F1CDx; Foundation Medicine, Inc., Cambridge, MA). Blood-based genomic profiling was undertaken on cell free DNA (cfDNA) extracted from plasma using the FoundationOne®Liquid CDx assay (F1LCDx; Foundation Medicine, Inc., Cambridge, MA). Both assays were performed in ISO accredited laboratories.

Next Generation Sequencing (NGS) was carried out using targeted high throughput hybridization-based capture technology to detect and report substitutions, insertions and deletions (indels), gene rearrangements/structural variants (SV), copy number alterations (CNA) and genomic signatures including microsatellite instability (MSI) and tumour mutational burden (TMB). TMB is a genomic signature that quantifies the frequency of somatic mutations in a patient's tissue or circulating tumour DNA [2]. Tissue-TMB (tTMB) is calculated based on synonymous and non-synonymous variants with an allele frequency of ≥5% while blood-TMB (bTMB) is calculated based on variants with an allele frequency of ≥0.5%.

Data was processed and analysed by Foundation Medicine into a curated clinical report (PDF), variant XML (eXtensible Markup Language) data files were also provided. Both the clinical report and variant XML data were uploaded to the eTARGET digital interface (https://upsmart.digitalecmt.com/etarget-3/). All CUPCOMP data was reanalysed, by Foundation Medicine, following an analysis pipeline update to include enhanced Comprehensive Tumour Fraction Scores (CTFS) (for detailed methods see [3]). In brief, the calculation of CTFS uses multi-parameter genomic features including aneuploidy-based copy number based estimation of tumour signal, somatic variant allele frequencies where there is little or no aneuploidy signal, fragment length information and removal of signal from Clonal haematopoiesis of Indeterminate Potential (CHIP) or germline variants. These updated files were used for any downstream retrospective, comparative analysis.

Whole Genome Sequencing of tumour tissue and germline control

For germline extractions 0.4 – 2 mL of blood from EDTA blood collection tubes had DNA extracted using the Chemagic Prime instrument according to manufacturer's instructions. DNA extraction from fresh frozen (FF) tissue was performed using the QIAGEN Tissue kit (QIAGEN, catalogue

number 953034) on the EZ1 XL instrument as per manufacturer's instructions. WGS was undertaken on extracted DNA by Source Bioscience (Source Biosciences UK Ltd, Nottingham, UK). DNA was quantified and quality assessed using Qubit double stranded DNA assay for concentration, Nanodrop for OD260/280 ratio and Agilent Tapestation for size distribution. Library preparation was undertaken according to internal Standard Operating Procedures with initial mechanical shearing to fragment the genomic DNA and Roche KAPA Hyper prep PCR free kit. All sequencing was performed using the Illumina NovaSeq 6000 instrument to generate reads of 2x 150bp to provide a total of 30x coverage for the germline sample and 75x coverage for the somatic sample. Bioinformatic analysis was performed and reported in Hypertext Markup Language (HTML) format generated. Central site uploaded the XML data files and HTML report to digital interface and results were extracted for analysis. In addition, all raw FASTQ and BAM files were transferred and stored within secure data-lake for future analysis and data sharing.

Retrospective genomic analysis

All blood and tissue Foundation Medicine data (XML format) were uploaded to a secure network drive (Cancer Research UK Manchester Institute, UK). Alteration and report data were extracted from tissue and updated blood XML files using an in-house R Script. Alteration data were extracted including nucleotide variants (SNVs; including small insertions/deletions (indels)), copy number alterations (CNA) and structural variants (SV) in addition to blood and tissue Tumour Mutation Burden (TMB), Microsatellite Instability (MSI), and Comprehensive Tumour Fraction Score (CTFS), where relevant. Data were converted into comma-separated values (CSV) and mutation annotation format (MAF) and pre-processed further for downstream OncoKb analysis[1] (see below). High-TMB was defined as ≥10 Mutations per Megabase (Muts/Mb) and applied for both blood and tissue. Correlative analysis of TMB scores was performed using a two-tailed Pearson correlation coefficient. For retrospective blood-based analysis, all baseline blood samples were used. In the absence of a baseline blood sample a progression blood sample was used. Variant Allele Frequency (VAF) analysis from blood-based testing was performed for SV and SNV alterations only.

Blood and tissue molecular alteration concordance

Concordance of mutational alterations was performed in patients where panel-based Next Generation Sequencing (NGS) from Foundation Medicine was performed in both tissue and blood. Concordance was calculated by the frequency of alterations (SNVs; including small insertions/deletions (indels)), copy number alterations (CNA) and structural variants (SV)) that

were found in both tissue and blood divided by the total unique alterations found across both modalities. This was calculated per patient and across all alterations found in the patients where both tissue and blood molecular profiling had been undertaken and evaluated by alteration type. Correlative analysis of alteration concordance against CTFS was performed using a two-tailed Pearson correlation coefficient.

Determining Oncogenicity and Actionability by OncoKB

Alterations were pre-processed and restricted to those whose Variant_Classification field was one of Frame_Shift_Del, Frame_Shift_Ins, Splice_Site, Translation_Start_Site, Nonsense_Mutation, Nonstop_Mutation, In_Frame_Del, In_Frame_Ins or Missense_Mutation. Alteration data were retrospectively passed through OncoKB to determine actionability and oncogenicity[1], this information was combined into MAF format for downstream analysis and visualisation using maftools [4] (v2.8.05) including generation of Oncoplot figures.

SUPPLEMENTARY REFERENCES

1 Chakravarty D, Gao J, Phillips SM et al. OncoKB: A Precision Oncology Knowledge Base. JCO Precis Oncol 2017; 2017.

2 Chalmers ZR, Connelly CF, Fabrizio D et al. Analysis of 100,000 human cancer genomes reveals the landscape of tumor mutational burden. Genome Medicine 2017; 9 (1): 34.

3 Rolfo CD, Madison RW, Pasquina LW et al. Measurement of ctDNA Tumor Fraction Identifies Informative Negative Liquid Biopsy Results and Informs Value of Tissue Confirmation. Clin Cancer Res 2024; 30 (11): 2452-2460.

4 Mayakonda A, Lin D-C, Assenov Y et al. Maftools: efficient and comprehensive analysis of somatic variants in cancer. Genome research 2018; 28 (11): 1747-1756.
