# Supplementary material for: A prospective national precision medicine trial evaluating the feasibility of blood-based molecular profiling in patients with Cancer of Unknown Primary (CUP-COMP)
Source: Br J Cancer. 2026 Jul 20;135(8):1255–66. doi: 10.1038/s41416-026-03519-6 (PMC13642309; doi:10.1038/s41416-026-03519-6)
Supplement: Supplementary file 2 [file 41416_2026_3519_MOESM3_ESM.pdf]

DocuSign Envelope ID: BF6EC156-05BF-4243-A38A-AE415088BB25

CUP-COMP

Carcinoma of Unknown Primary (CUP):

A comparison across tissue and liquid biomarkers

Protocol:	Version 3.0 dated 01.02.2023
Chief Investigator:	Dr Natalie Cook
Sponsor:	The Christie NHS Foundation Trust
Sponsor Reference:	CFTSp188
Funder:	Innovate UK
IRAS Number:	284749
REC Reference:	20/YH/0305

Protocol Version History	
Change to end of study definition. Reduction of the lower recruitment target. Removal of post-baseline fresh tissue sample collection. Amended SAE reporting as per sponsor SOP. Clarification of protocol to reflect previous non-substantial amendments.	Version 3.0 Dated 01/02/2023
Clarification of biopsy collection time point	Version 2.1 Dated 21/10/2021
Addition of retrospective cohort of archival tissue	Version 2.0 Dated 03/02/2021
REC response	Version 1.1 Dated 13/11/2020
Original Protocol	Version 1.0 Dated 07/10/2020

Signature Page

Carcinoma of Unknown Primary Site (CUP): A comparison across tissue and liquid biomarkers

This document describes the CUP-COMP study and provides information about procedures for entering participants into it. The protocol should not be used as a guide for the treatment of participants outside of the study.

The undersigned confirm that the following protocol has been agreed and accepted and that the Chief Investigator agrees to conduct the study in compliance with the approved protocol and will adhere to the principles outlined in the ICH Good Clinical Practice guidelines, UK Policy Framework for Health and Social Care and the Sponsor's SOPs.

Every care was taken in drafting this protocol, but corrections or amendments may be necessary. These will be circulated to all Investigators in the study as required.

For an on behalf of the Study Sponsor:

Clare Griffin, Research Integrity and Governance Manager

Signature: DocuSigned by: Clare Griffin 98D0B6F8A3D9430...

Date: 22-Mar-2023

Chief Investigator:

Dr Natalie Cook, Senior Clinical Lecturer and Honorary Consultant in Medical Oncology

Signature: DocuSigned by: Natalie Cook 88F8C62E772744A...

Date: 22-Mar-2023

Key Study Contacts

Role	Name and Position	Contact details
**Chief Investigator**	Dr Natalie Cook<br>Senior Clinical Lecturer in Experimental Cancer Medicine & Honorary Medical Oncology Consultant	The Christie NHS Foundation Trust,<br>Wilmslow Road, Manchester, M20 4BX<br>Phone: 0161 918 7672 (Secretary)<br>Email: Natalie.cook17@nhs.net
**Co-Investigator**	Dr Kai Keen Shiu<br>Consultant Medical Oncologist & Honorary Associate Professor	University College London Hospitals NHS Foundation Trust,<br>250 Euston Road, London, NW1 2PG<br>Phone: 020 3456 7890<br>E-mail: k.shiu@ucl.ac.uk
**Co-Investigator**	Dr Sonali Dasgupta<br>Consultant Medical Oncologist	Velindre University NHS Trust,<br>Velindre Road, Cardiff, CF14 2TL<br>Phone: 029 2061 5888 ext. 6372<br>Email: Sonali.Dasgupta@wales.nhs.uk
**Co-Investigator**	Dr Eliyaz Ahmed<br>Consultant in Medical Oncology & Honorary Clinical Lecturer	The Clatterbridge Cancer Centre NHS Foundation Trust,<br>Clatterbridge Road, Bebington, Wirral CH63 4JY<br>Phone: 0151 5565031<br>Email: e.ahmed@nhs.net
**Co-Investigator**	Dr Louise Medley<br>Consultant Medical Oncologist	Torbay and South Devon NHS Foundation Trust<br>Torbay Hospital, Lowes Bridge Torquay, TQ2 7AA<br>Phone: 01803 655643<br>Email: louise.medley@nhs.net
**Co-Investigator**	Dr Sally Clive Consultant Medical Oncologist	Edinburgh Cancer Centre,<br>The University of Edinburgh<br>Western General Hospital<br>Crewe Road South, Edinburgh EH4 2XR<br>Phone: 0131 537 2263<br>Email: sally.clive@nhslothian.scot.nhs.uk
**Co-Investigator**	Dr Tania Tillett<br>Medical Oncology Consultant	Royal United Hospitals Bath NHS Foundation Trust<br>Combe Park, Bath, BA1 3NG<br>Phone: 07970 796440<br>Email: t.tillett@nhs.net

Role	Name and Position	Contact details
**Co-Investigator**	Dr Claire Mitchell<br>Consultant Medical Oncologist	The Christie NHS Foundation Trust,<br>Wilmslow Road, Manchester, M20 4BX<br>Phone: 0161 446 3606<br>Email: Claire.mitchell34@nhs.net
**Co-Investigator**	Dr Pedro Oliveira	The Christie NHS Foundation Trust,<br>Wilmslow Road, Manchester, M20 4BX<br>Phone: 0161 446 3275<br>Email: Pedro.Oliveira1@nhs.net
**Academic Collaborators**	Dr Alicia-Marie Conway,<br>Dr Dominic Rothwell,<br>Professor Caroline Dive	Cancer Research UK Manchester Institute Cancer Biomarker Centre, The University of Manchester<br>Block 13S58, Alderley Park, SK10 4TG
**Study Manager - Commercial**	Dr Kelly Warrington,<br>Medical Project Lead: Personalised Healthcare	6 Falcon Way,<br>Shire Park, Welwyn Garden City, AL7 1TW<br>Phone: 07717580921<br>Email: kelly.warrington@roche.com
**Study Manager - Academic**	Dr Matthew Concannon,<br>Research Project Manager,<br>Experimental Cancer Medicine Team (ECMT),<br>Research and Innovation Department	2nd Floor, Oak Road Treatment Centre,<br>The Christie NHS Foundation Trust,<br>Wilmslow Road, Manchester, M20 4BX<br>Phone: 0161 918 2473<br>Email: the-christie.cup-comp@nhs.net
**Sponsor Representative**	Dr Clare Griffin<br>Research Integrity and Governance Manager,<br>Research and Innovation Department	Oglesby Cancer Research Building,<br>The Christie NHS Foundation Trust,<br>Wilmslow Road, Withington, M20 4BX.<br>Phone: 0161 918 7432<br>Email: the-chrisite.sponsoredresearch@nhs.net

DocuSign Envelope ID: BF6EC156-05BF-4243-A38A-AE415088BB25

Table of Contents

1.0 Abbreviations 10

2.0 Study Summary 12

3.0 Study Schema 13

4.0 Background 15

4.1 Background 15

4.2 Rationale 20

4.3 Overall Risk/Benefit 21

5.0 Research Question 24

5.1 Primary Objective 24

5.2 Secondary Objective 24

5.4 Primary Outcome Measure 24

5.5 Secondary Outcome Measure 25

5.7 End of Study Definition 26

6.0 Study Procedures 27

6.1 Overview 27

6.2 Study Schedule 28

7.0 Recruitment and Selection of Study Participants 36

7.1 Informed Consent 37

7.2 Participant Selection Criteria 37

7.3 Participant Inclusion Criteria 39

7.4 Participant Exclusion Criteria 39

7.5 Study Payment 40

7.6 Participant Registration 40

7.7 Discontinuation of Participants 40

DocuSign Envelope ID: BF6EC156-05BF-4243-A38A-AE415088BB25

7.8 Withdrawal of Participants 41

8.0 Statistics and Data Analysis 41

8.1 Sample Size 41

8.2 Recruitment Rate and Interim Analysis 42

9.0 Data Management 42

9.1 Case Report Forms 44

9.2 Data Storage 44

9.3 Access to Data 44

9.4 Confidentiality 45

9.5 Archiving 45

10.0 Administrative and Regulatory Details 46

10.1 Study Sponsor 46

10.2 Peer Review 46

10.3 Funding Body 46

10.4 Ethics Approval 46

10.5 Participant and Public Involvement 47

10.6 Study Monitoring 47

10.7 Protocol Compliance 48

10.8 Safety Reporting 49

10.8.1 Terms and Definitions 49

10.8.1.1 Adverse Events 49

10.8.1.2 Serious Adverse Events 49

10.8.2 Notes on Adverse Event Inclusions and Exclusions 49

10.8.3 Evaluating Adverse Events 50

10.8.4 Reporting of Serious Adverse Events 50

10.9 Study Oversight 51

10.10 Protocol Amendments 51
10.11 Liability and Indemnity 52
10.12 End of Study Notification 53
10.13 Study Summary Report 53
10.14 Publication Policy 53
11.0 References 55

List of figures

Figure 1: Study schema
Figure 2: ESMO Guidance on clinical management of CUP
Figure 3: The overall proposed pathway for CUP patients who enrol onto this study
Figure 4: Sample pathway flowchart

List of tables

Table 1: Proposed treatments for CUP patients with a defined subset
Table 2: Schedule of events
Table 3: Sample collection schedule of events

List of appendices

Appendix 1: Summary of ESMO guidelines
Appendix 2: Serious Adverse Event reporting form

1.0 Abbreviations

AcoRD	Attributing the costs of health and social care Research and Development
AE	Adverse Event
cfDNA	Circulating Free DNA
CI	Chief Investigator
CRF	Case Report Form
CSG	Clinical Studies Group
CTC	Circulating Tumour Cells
ctDNA	Circulating Tumour DNA
CUP	Carcinoma of Unknown Primary
ESMO	European Society for Medical Oncology
GCP	Good Clinical Practice
GDPR	General Data Protection Regulation
HTA	Human Tissue Act
ID	Identification
LDH	Lactate Dehydrogenase
LPLV	Last Patient Last Visit
MCRC	Manchester Cancer Research Centre
MDT	Multidisciplinary Team
MTB	Molecular Tumour Board
NIHR	National Institute for Health Research
NHS	National Health Service
PIS	Participant Information Sheet
REC	Research Ethics Committee
RNA	Ribonucleic Acid
SAE	Serious Adverse Event
SOP	Standard Operating Procedure
TMG	Trial Management Group

USM	Urgent Safety Measure
WGS	Whole Genome Sequencing

2.0 Study Summary

**Title**	Carcinoma of Unknown Primary Site (**CUP**):<br>A **comp**arison across tissue and liquid biomarkers
**Acronym**	CUP-COMP
**Study Design**	An exploratory, multi-centre, clinical sample collection study
**Study Participants**	Adult patients with a confirmed diagnosis of CUP (please refer to section 7 for full eligibility criteria)
**Planned Sample Size**	Approximately 100-140 patients
**Planned Study Duration**	18 months (+12 months recruitment [delayed study start due to COVID-19 and lower than expected recruitment rate] + 12 months for follow up data collection and sample analysis)
**Study Objectives**	• Assessment of genomic sequencing (both in tissue and blood) for the diagnosis and treatment stratification in patients with CUP including a comparison of the effectiveness of tissue and blood based biomarkers<br>• Collection of evidence to further develop technology that predicts an individual's response to a treatment.<br>• Development of innovative systems of clinical data capture in patients with CUP
**Study Endpoints**	• Sequence tumour tissue and circulating tumour DNA from approximately 100-140 patients with CUP in order to evaluate the activation state of various oncogenic pathways and improve treatment stratification approaches<br>• To establish a genomic reporting mechanism whereby clinically relevant and potentially ‘actionable’ abnormalities found during sequencing/molecular characterisation can be reported to patients<br>• To build on close collaborations between the NHS Hospitals treating patients with CUP, and develop a data collection repository within the NHS<br>• Work with external collaborators to progress basic and translational research models to investigate biomarkers to treatment in CUP and key biological drivers of this disease

3.0 Study Schema

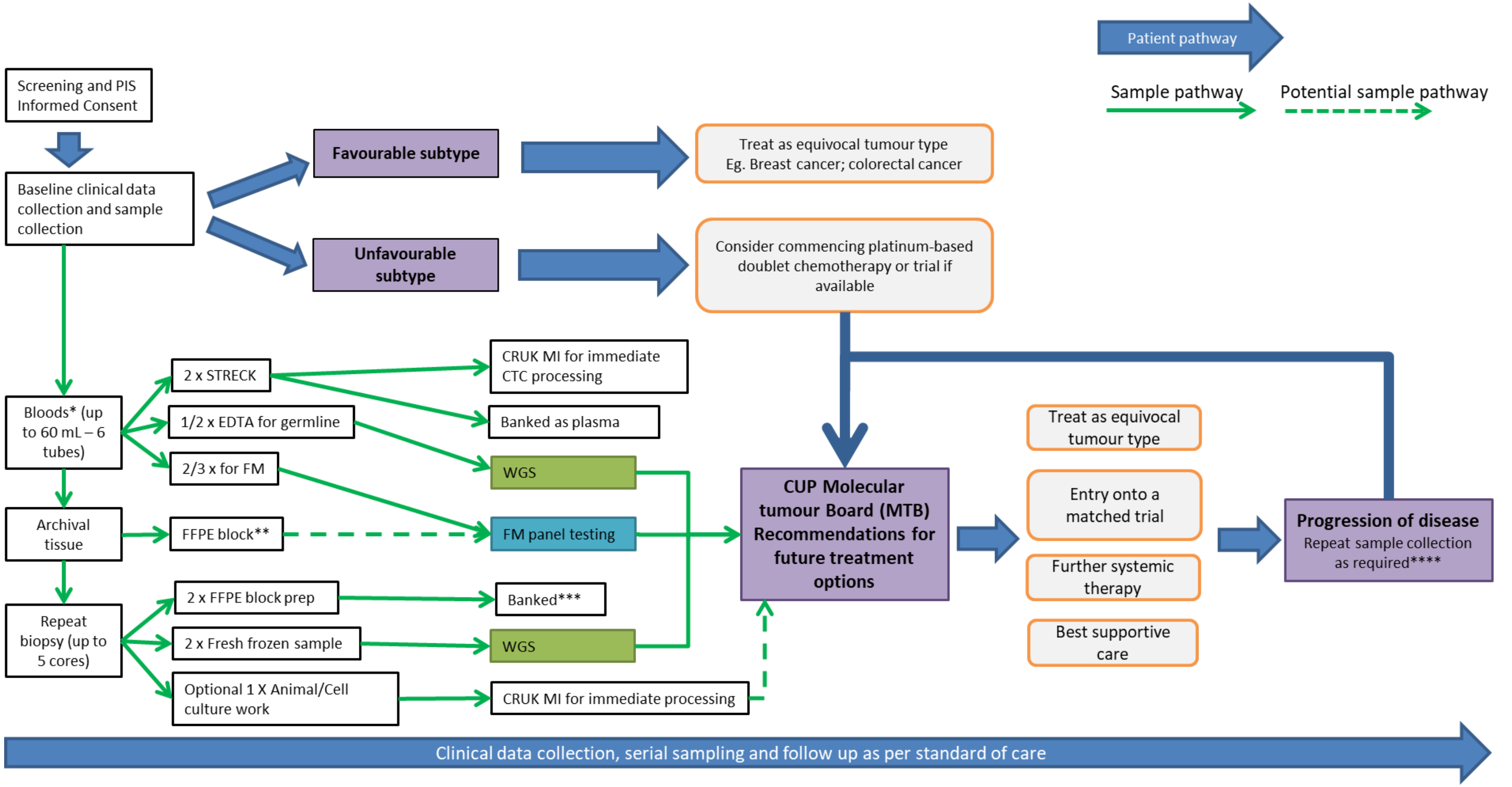

Figure 1: Overall patient and sample pathway flow for CUP-COMP.

*One tube of blood is approximately 10mls.

**FFPE blocks will only be sent for FM panel testing where a fresh biopsy is not safe to undertake and where there is enough tissue remaining

***Extra FFPE blocks will be banked at the Christie NHS Foundation Trust

****Repeat FM blood collection available.

CRUK MI: Cancer Research UK Manchester Institute

FM: Foundation Medicine

PIS: Participant information sheet

mL: millilitres

WGS: whole genome sequencing

4.0 Background

4.1 Background

CUP

Cancers of unknown Primary (CUP) are an important but historically under-researched clinical entity. CUP remains an unmet clinical and research need. It is the 6th leading cause of cancer death in the UK and prognosis is poor with a median survival of 6-9 months(Cancer Research UK, 2020), Patients with CUP have metastatic cancer at diagnosis but standard investigations fail to identify a primary site of origin of the tumour (Massard, Loriot and Fizazi, 2011; Fizazi *et al.*, 2015). Patients generally have a very poor prognosis and limited treatment options due to the difficulties in determining the origins of the cancer through usual examination of the tumour tissue. No clear risk factors have been identified for CUP to date (Pavlidis and Fizazi, 2009) with most patients presenting with extensive metastatic disease. Due to these circumstances, early detection of patients with CUP is not possible.

A clinical algorithm for the management of CUP patients has been developed by The European Society for Medical Oncology (ESMO) (Figure 2: Management of patients with CUP. Taken from ESMO guidance). If there is suspicion of a primary cancer, yet following clinical examination, immunohistochemistry, radiological review and laboratory review a primary cannot be found, treatment would be recommended based on known available site specific therapies for the proposed cancer type (Fizazi *et al.*, 2015). Patients where a primary cannot be found or suspected are classified into favourable prognosis CUP or poor prognosis CUP, based on clinico-pathological features. The subsequent treatment regimen initiated is based on this (Figure 2).

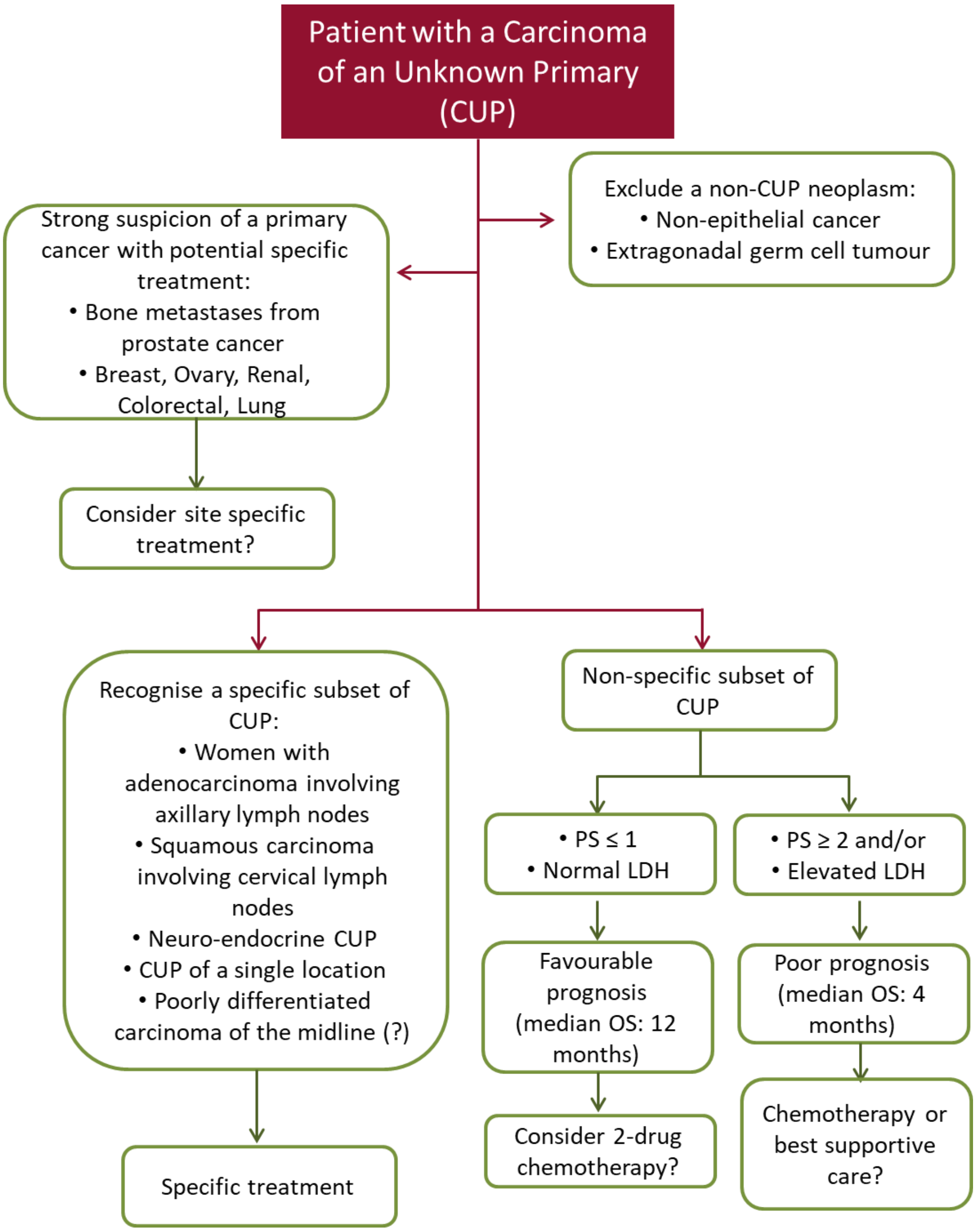

Figure 2. ESMO Guidance on clinical management of CUP. Adapted from (Fizazi *et al.*, 2015).

Favourable prognosis CUPs

Defined clinic-pathological subsets of CUP are observed in 15-20% of patients; these patients have chemo-sensitive tumours. Treatment is dependant on individual subtypes, and where the disease is managed similarly to those with a potential equivalent tumour, long term disease control can be achieved (Table 1 – Fizazi *et al.* 2015).

Table 1: Proposed treatments for patients with a CUP diagnosis, with a defined subset, adapted from Fizazi *et al*. 2015

CUP subtype	Proposed treatment
Poorly differentiated neuroendocrine carcinomas of an unknown primary	Platinum + etoposide combination chemotherapy
Well-differentiated neuroendocrine tumour of unknown primary	Somatostatin analogues, combo chemotherapy, everolimus
Peritoneal adenocarcinomatosis of a serous papillary histological type in females	Optimal surgical debulking followed by platinum–taxane-based chemotherapy
Isolated axillary nodal metastases in females	Axillary nodal dissection, mastectomy or breast irradiation and adjuvant chemotherapy and hormone therapy
Squamous cell carcinoma involving non-supraclavicular cervical lymph nodes	Neck dissection and/or irradiation of bilateral neck and head–neck axis. For advanced stages induction chemotherapy with platinum-based combination or chemoradiation
CUP with a colorectal IHC (CK20+ CDX2+ CK7−) or molecular profile	Systemic treatment used for colorectal cancer
Single metastatic deposit from unknown primary	Resection and/or RT ± systemic therapy
Men with blastic bone metastases or IHC/serum PSA expression	Androgen deprivation therapy ± RT

5-FU, 5-fluorouracil; MRI, magnetic resonance imaging; IHC, immunohistochemistry; PSA, prostate-specific antigen; RT, radiotherapy; CK, cytokeratin

Poor Prognosis CUPs

80-85 % patients with a diagnosis of CUP have a poorer prognosis due to limited sensitivity to treatments; they belong to the non-specific subset of CUP. Patients in this group are divided into two prognostic groups;

1. Good performance status of 0-1, normal lactate dehydrogenase (LDH) with a median life expectancy of 1 year
2. Poor performance score, elevated LDH, median life expectancy of approximately 4 months. These patients are more likely to be treated with combination chemotherapy regimens such as cisplatin and gemcitabine or cisplatin/carboplatin and paclitaxel (Fizazi *et al.*, 2015).

No specific chemotherapy regimen has been identified as superior to others in poor prognosis CUPs despite multiple small number non-randomised studies. Agents investigated include platinum, gemcitabine, taxane and irinotecan (Culine *et al.*, 2003; Golfinopoulos *et al.*, 2009; Hainsworth *et al.*, 2010; Gross-Goupil *et al.*, 2012). The standard first line treatment for this patient population is usually platinum based doublet combination (Fizazi *et al.*, 2015). To date there is no evidence to support the addition or use of targeted therapies from the small number of phase II studies that have evaluated them.

Molecular biomarkers

Liquid and tissue biopsies offer an opportunity to detect tumour molecular and immune characteristics. In the blood (liquid) this includes but is not limited to circulating tumour DNA (ctDNA) and circulating tumour cells (CTCs). These tests have the potential to help determine primary tissue of origin or biomarkers that are predictive of response to certain therapies, including immunotherapy (Conway *et al.*, 2019). However even though we could characterise CUP tumours by these genetic changes it is unclear if treating patients based on these predictions improve survival. Currently routine tissue-of-origin predictions are not recommended in CUP because of the lack of evidence to support improved clinical outcomes.

There is emerging data investigating the area of liquid biopsy molecular testing in CUP (Kato *et al.*, 2017). Most previous data comes from tumour tissue profiling despite challenges with access to tissue of sufficient quality and quantity. Small diagnostic biopsies have often undergone multiple immunohistochemistry stains prior to diagnosis and repeat biopsies are often required. Liquid biopsies have the potential to overcome some of the inherent limitations of tissue biopsy; reflecting the whole tumour burden and enable less invasive and serial sampling (Conway *et al.*, 2019).

A variety of algorithms have been used to build predictive models from liquid biopsies, including support vector machines, generalised linear models, linear discriminate analysis, decision trees, random forests,

and unsupervised clustering (Ko *et al.*, 2018), however these methods have been primarily used to either detect the presence of a cancer or classify the cancer present (Potie *et al.*, 2019).

Future research and treatment

Despite significant advances and new drug development for other cancers, there are no approved targeted therapies or immunotherapies for CUP, and currently no UK approved funded molecular genomic tests. Limited progress has been made due to the lack of research and funding into CUP, and therefore novel approaches to improve prognosis are desperately needed. Examining the genetic characteristics of CUP tissue and blood biomarkers could predict response to existing or new therapies, and has already been studied in other cancer types (Dahlman *et al.*, 2012; Shaw *et al.*, 2014; Grigoriu, Berghmans and Meert, 2015; Lu *et al.*, 2018).

There have been previous studies investigating site-specific treatment of CUP on the basis of prediction of the primary site by molecular or expression-based profiling (Fizazi *et al.*, 2019; Hayashi *et al.*, 2019). One of the main issues with these studies has been lack of access to novel targeted therapeutics leading to disappointing efficacy signals in the treatment arms. Recently a large global phase II randomised study opened for patients with poor prognosis CUP (MX39795) (ClinicalTrials.gov identifier: NCT03498521, https://clinicaltrials.gov/ct2/show/NCT03498521). This study aims to compare the efficacy and safety of targeted therapy or cancer immunotherapy guided by molecular testing versus standard platinum-based chemotherapy. A significant number of compounds have been made available, including newly approved TRK inhibitors (Drilon, 2019). Results from this study are expected in approximately June 2023. There are also a number of studies investigating immunotherapy in patients with CUP, some with early encouraging results (Tanizaki *et al.*, 2020). It is reported a large number of patients with CUP have therapeutically actionable mutations in their tumours (Ross *et al.*, 2015; Kato *et al.*, 2017) however there remains a lack of prospective data matching these patients to therapeutic options so the results of these trials are eagerly awaited.

There is also limited understanding of the diversity of genomic and epigenomic features contributing to CUP pathogenesis and progression outside of clinical sequencing panels. The influence of such features has not been widely assessed from existing clinical trial data, nor has a clinical study been designed to identify novel genomic and epigenomic biomarkers. Therefore, there is significant value in pursuing

approaches capable of identifying clinically actionable biomarkers, both for patient treatment and scientific understanding of CUP.

4.2 Rationale

CUP is an under-researched entity with a poor prognosis and limited treatment options. Treatment options are limited due to the lack of positive biomarkers and difficulties in determining tissue of origin of the tumour. Within solid tumour types of known tissue of origin newer therapies, including targeted therapy and immunotherapies, have dramatically improved survival in tumour types with previously poor prognosis. The role of these novel therapies in CUP has not been well investigated but it is likely that a proportion of patients with CUP may benefit from such treatments.

The global CUPISCO study (clinicaltrials.gov identifier: NCT03498521, https://clinicaltrials.gov/ct2/show/NCT03498521) may be able to answer some of these questions however this study is only recruiting specific sub-groups of CUP who have not received prior treatment, and has extremely strict eligibility criteria, with the potential for a delay in treatment start date for patients. Therefore there are many patients diagnosed with CUP that would not be able to participate in this study. Most UK patients with CUP only have access to first line doublet chemotherapy with limited response rates and no standard second line therapy. They are usually ineligible for experimental cancer trials as CUP is never called out as a tumour type of interest. With identification of tissue of origin or markers that are predictive of response to therapies we would hope that this could improve clinical outcomes, and even entry to clinical trials. Studying the molecular characteristics of CUP tumour tissue and liquid biopsies will help to understand the heterogeneity of the tumours and may also reveal novel therapeutics for a subset of patients.

Access to reliable tissue is difficult in patients with CUP as often a small biopsy has undergone multiple immunohistochemistry stains prior to a diagnosis of CUP. This makes researching and investigating CUP tumours difficult. Patient's symptoms, blood test results, endoscopic evaluation, radiological imaging and pathology results are all currently used as standard diagnostics to make clinical decisions for patients with CUP. To date there have been limited studies investigating molecular genomics in patients with CUP, and as a result we have inadequate evidence to evaluate whether genomic profiling has added value over and above the standard diagnostics provided in the NHS.

We have formed a collaboration that spans Academia, the NHS, large and small industrial enterprises and the charitable sector in order to compare genomic assays in the diagnosis and treatment stratification in CUP (**CUP-COMP**). This study unites seven NHS Hospitals with research interests in CUP, a large Cancer

Research UK centre, a global pharmaceutical company with a heritage in oncology and diagnostics (Roche), expert data management (ConcR) alongside patient representation and advice from the CUP Foundation Jo's Friends (https://cupfoundjo.org/). This is a group of collaborators who share a vision of working effectively and efficiently together in order to progress the diagnosis and treatment choices for patients with CUP.

In order to make further progress for patients with CUP we need access to extra tissue and blood samples for molecular testing, and also for research initiatives. We want to understand the profile of CUP tumours in the material we collect. This project will enrol patients across all subtypes of CUP, gathering evidence for the multitude of questions that remain unanswered. The genomic and longitudinal clinical data that will be collected would be the first dataset to include multi-omic and clinical data and could be used to drive additional research in the UK. It would address an area of high unmet need and has the potential to change clinical care. It has the full support of the National Cancer Research Institute (NCRI) Clinical Studies Group (CSG) CUP workstream (http://csg.ncri.org.uk/ncri_groups/ncri-living-with-and-beyond-group/).

Key Objectives

- Assessment of genomic sequencing (both in tissue and blood) for the diagnosis and treatment stratification in patients with CUP including a comparison of the effectiveness of tissue and blood based biomarkers
- Development of novel computational methods to identify tissue-of-origin biomarkers and subsequently predict patient-specific treatment response
- Development of innovative systems of clinical data capture in patients with CUP

4.3 Overall Risk/Benefit

This study has been classified as somewhat higher than the risk of standard of medical care.

Risks

1) **Data/ Confidentiality:**

General risks associated with data collection apply. Personal patient information will be protected through pseudo- anonymisation using a unique patient identifier that will be assigned upon enrolment onto study. All data will be handled in line with the Data Protection Act 2018 and

DocuSign Envelope ID: BF6EC156-05BF-4243-A38A-AE415088BB25

General Data Protection Regulation. Data will be stored on password protected electronic databases and source data will be stored in locked research offices within the NHS. Data will be pseudo- anonymised before transfer to external collaborators (ConcR and CRUK Manchester Institute) using a CUP-COMP specific trial ID number. Data held by ConcR will be encrypted at rest on a secure computing platform. Calculations on the data will be performed within a computational environment with restricted and audited data export facilities. All access and use of the data held by ConcR will be logged and audited.

There are some specific risks related to genomic data, please see section 9.0 for detail.

2) Procedural:

- There are some risks associated with biopsy collection. Risks are dependent on the exact procedure performed and will be discussed with the patient based on individual circumstances. Separate consent will be obtained at the time of sample collection which is part of standard trust policy. General biopsy risks include pain, inflammation, bleeding, swelling, scarring, or infection. Biopsies would be taken under ultrasound or CT guidance.
- Minor risks are associated with blood sample collection such as bruising and pain. These samples will be taken by appropriately trained staff at the clinical sites according to site delegation logs.
- Genetic research may result in psychological distress if results show other previously unknown conditions (e.g hereditary conditions). All participants will receive the appropriate information about this at the time of consent and will be given the option to not have any such results disclosed. If a patient does choose to have any incidental findings reported back to them, they will be referred to a genetic counselling service as appropriate.

Benefits

- Although the objectives of this study are not directly therapeutic and therefore may not have a direct benefit for all participants, it is anticipated that the results from the tests may help direct some participants onto a suitable treatment option. There is also the benefit of increased knowledge about molecular changes in their cancer which some patients may also find useful.

- The data collected from this project will help to develop the understanding of CUP, in particular the discovery of clinically relevant biomarkers to assist with the diagnosis and treatment stratification for patients, thus aiming to improve access to targeted therapies for this patient group with currently limited options. This information will be made widely available following relevant publications.
- Assessment of the benefit of using WGS and ctDNA profiling as part of this protocol has the potential to benefit patients with CUP in the future. This group is currently not represented or funded as part of the National Genomics test directory.

5.0 Research Question

5.1 Primary Objective

- The primary aim of this protocol is to establish a mechanism to assess and compare genomic sequencing (in tissue and blood) for the diagnosis and treatment stratification for patients with CUP

5.2 Secondary Objective

Secondary objectives include;

- To collect and analyse samples from patients with CUP for future translational research analysis
- To facilitate and establish a pathway in order to use the clinically significant sequencing/profiling data to assist clinicians in directing delivery of personalised cancer therapy to patients with CUP
- Develop systems of clinical data capture in patients with CUP

5.3 Exploratory Objective

- To develop and validate a technology to predict patient response to treatment informed by clinical and –omic information
- Identification of any tissue-of-origin specific biomarkers
- Collect and evaluate evidence to develop technology that predicts an individual's response to a treatment in CUP

5.4 Primary Outcome Measure

The primary outcomes measures are;

- To sequence tumour tissue and circulating tumour DNA from approximately 100- 140 patients with CUP in order to evaluate the activation state of various oncogenic pathways and improve treatment stratification approaches

- To establish a genomic reporting mechanism whereby clinically relevant and potentially 'actionable' abnormalities found during sequencing/molecular characterisation can be reported to patients

5.5 Secondary Outcome Measure

- To obtain archival tumour specimens, fresh tissue and sequential blood samples from approximately 100 – 140 patients with CUP
- The comparative assessment of alternative assay methods (such as ctDNA) in parallel with whole genome sequencing in the diagnosis and treatment stratification for patients with CUP
- To collect samples for future analysis for additional molecular characterisation techniques for detection of actionable aberrations. Examples include RNA sequencing, IHC, proteomics, methylation, analysis of immuno-biomarkers
- To perform whole genome sequencing in patients where enough tumour tissue is available to explore novel predictive and resistance biomarkers
- To build on close collaborations between the NHS CUP Hospitals, and external collaborators to progress basic/translational research models to investigate biomarkers to treatment in CUP and key biological drivers of this disease.
- Collect evidence investigating the most effective way to stratify patients with CUP onto treatment.
- To understand whether liquid biomarkers in CUP can be used to stratify patients and give prognostic information
- Develop a data collection repository within the NHS for patients with CUP

5.6 Exploratory Outcome Measure

- Develop innovative systems of clinical data capture (Concr) to include molecular tests, follow up and outcomes on all patients. This evidence could be used to update treatment pathways and clinical guidelines and to support a NICE technical assessment of the value of genomic testing in patients with CUP

- Implementing Concr algorithms to understand how they performs versus health care professionals decisions

5.7 End of Study Definition

Unless early termination is required, the end of study is defined as the end of samples analysis. Recruitment will end on 16th February 2023. Follow up will end on 16th February 2024. Sample analysis will end on 31st March 2024.

6.0 Study Procedures

6.1 Overview

This is an exploratory multi-centre research project with the aim of discovering potential clinically useful biomarkers to aid the diagnosis and management of patients with CUP. We plan to offer sequencing of tumour tissue and ctDNA to approximately 100-140 patients with CUP, referred to selected centres across the UK. We hope to optimise CUP patient pathways and assess the feasibility of establishing a personalised cancer medicine approach for patients with CUP. We propose to collect fresh tumour tissue and blood samples from patients recruited. We will analyse DNA, RNA and/or other molecular aberrations in blood and tumour samples from these patients as well as germline DNA to allow the identification of key genomic and/or transcriptional changes.

Study participants will be identified from local CUP MDT discussions. These patients will have been referred to the CUP MDT and should be considered for standard treatment approaches. Members of the study team will identify eligible participants who voluntarily agree to be enrolled in this protocol. The process of informed consent will include counselling about risks and benefits of genomic sequencing results in which patient can choose a priori whether to receive the result of genetic analysis.

Biospecimens will be collected from patients who either undergo a standard of care procedure (e.g. tumour biopsy/ drainage of effusion) OR medically fit patients with accessible tumour who consent to undergo a fresh tumour biopsy for research purposes (using ultrasound or CT guidance). Archival tumour blocks from previous diagnostic biopsies may also be obtained for the purpose of this study. Additionally sequential blood samples will be collected to investigate biomarkers and resistance mechanisms to therapy. We intend in the future for all such analyses to be obtained through minimally invasive sample collection and this protocol will enable us to optimise and validate methods to achieve this.

Each collected biospecimen will be pseudo-anonymised and assigned with an identification (ID) number, which will be used by laboratory staff. Links between study ID number and clinical data will be held within secure, password-protected computers. Only key personnel with the need to link biomarker data with clinical data for clinical decision-making will have access to this database. Samples will be distributed to analysing laboratories in accordance with participating sites standard-operating procedures. Upon completion of analyses or at the end of the study, any leftover samples will be returned to Manchester

Genomics Laboratory, MCRC biobank facilities or their local institution and stored according to the biobank ethical approvals and guidelines.

6.2 Study Schedule

Table 2: Schedule of events

	Baseline	Progression [1]	Follow up 6 months (from baseline)	Follow up 12 months (from baseline)
Patient consent	X			
Medical History	X			
Blood Sample[a]	X	X[b]		
Archival tumour tissue retrieval[a]	X			
Fresh biopsy[a]	X			
Clinical data collection[c]	X	X	X	X
SAE reporting[d]		X	X	X

[1] Please only collect samples and data at the first treatment progression within the 12 month follow up period.

[a] Please see section 6.2.2 for detail on sample collection.

[b] Serial blood sample to be collected at one time point. Please see section 6.2.2. for detail on sample collection

[c] Please see section 9.0 for detail on clinical data collection

[d] Please see section 10.8 for detail on SAE reporting

The overall proposed pathway for patients with CUP who enrol onto this study is represented in Figure 3.

.

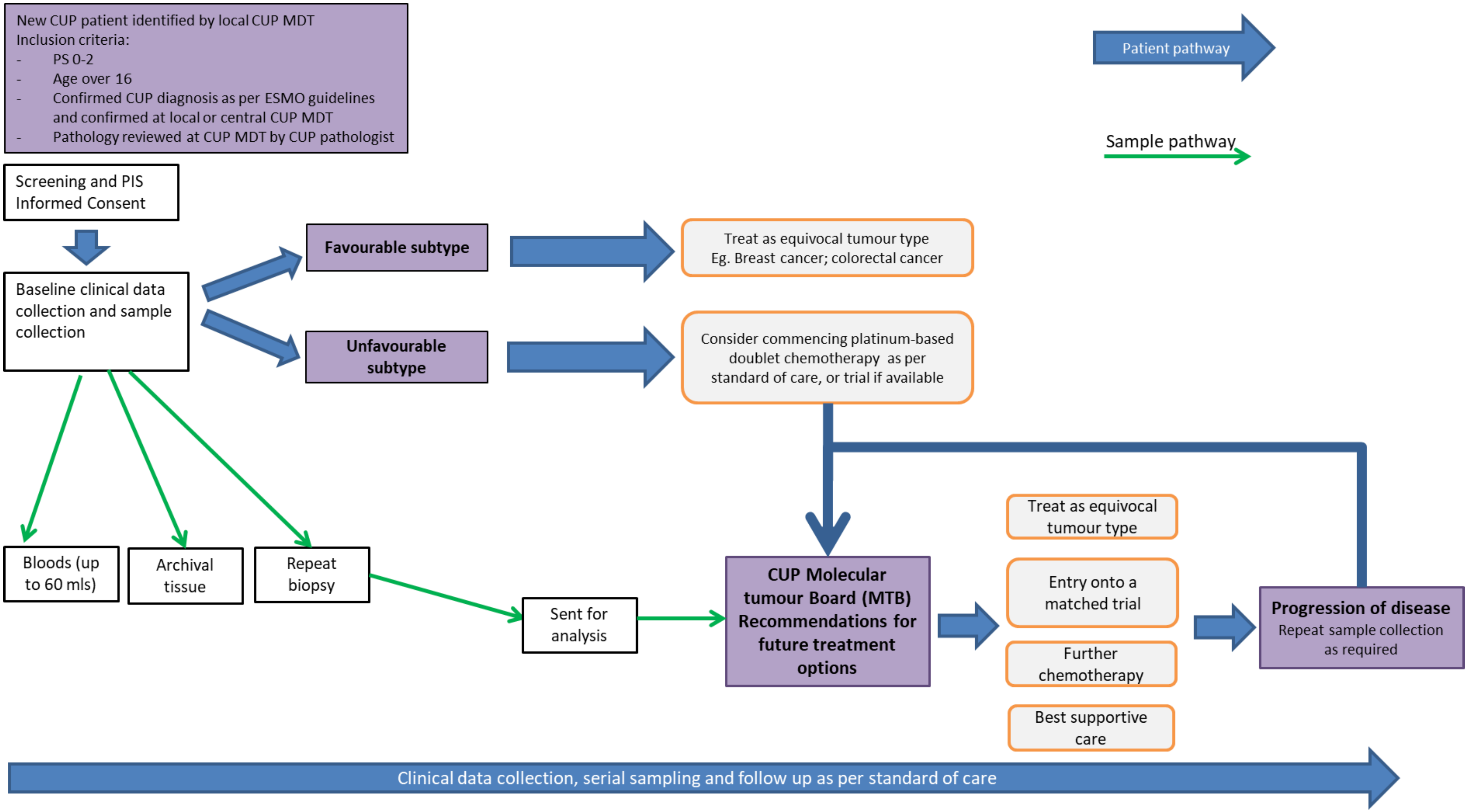

Figure 3: Patient pathway for CUP-COMP.

PIS: Participant Information Sheet, MDT: Multidisciplinary Team

6.2.2 Sample Collection

Once patients are consented to the study their archival tissue will be reviewed for tumour quantity at local CUP MDTs. Samples with adequate tissue will be analysed according to procedures below. If there is a lack of adequate tissue patients will be required to undergo a new tumour biopsy where feasible and safe to do so. This is often performed as standard of care in patients without a confirmed diagnosis of CUP, however this may also be performed in the context of this study for research purposes for which study specific consent will be obtained.

Samples will be collected as shown in table 3. The pathway of the samples is in figure 4.

Table 3: Sample collection schedule

	Baseline	Progression
Blood sample collection	X [a]	X [b]
Archival tumour block retrieval [c]	X	
Fresh tissue collection	X [d,e]	
Surplus tissue collection for animal model/cell culture research [f]	X	

[a] Up to 60ml blood will be collected for circulating tumour DNA (ctDNA), germline DNA, circulating tumour cells (CTCS), plasma and serum.

[b] Up to 17ml blood will be collected for ctDNA testing.

[c] Archival tumour blocks from a prior biopsy will be retrieved/requested. Samples will be sent for testing if no fresh tissue collection is to be performed and/or where FM blood tests are cancelled by the supplier for quality control reasons.

[d] Surplus fresh tumour tissue if undergoing a standard of care procedure where there is leftover tissue for research purposes. This may include (for example) biopsy, ascitic fluid and/or pleural effusions if being drained as a part of a clinically indicated procedure

[e] Fresh tumour tissue from an eligible patient willing to undergo a tumour biopsy of an accessible lesion for the purposes of this study. This can only be performed at the baseline timepoint.

[f] Optional for patients who consent. If surplus tissue available then a sample of this could be acquired to implant in a mouse or other relevant animal model/pre-clinical cell culture model

If patients are undergoing a biopsy for standard-of-care purposes or as part of a clinical trial, we will request patient's consent for surplus samples. Research biopsies for this protocol will only be requested where no other biopsies are planned for the individual. Patients will be subjected to a maximum of one research specific

biopsy at baseline only on this study. The biopsy should be performed prior to any new treatment where possible.

For each biopsy up to 5 tissues cores will be collected for

- Fresh frozen sample for Whole Genome Sequencing (WGS) (x2)
- Formalin fixed paraffin embedded (FFPE) block preparation (x2)
- Animal implantation/cell culture work where patients have provided explicit consent to do so and where there is excess tissue that could be used for this purpose

If less than 3 cores are available, samples will be prioritised according to the main research question for that individual. Samples will be sent to collaboration research laboratories at Penzburg, Germany (or Boston, USA as a back-up) for ctDNA analysis and the Manchester Genomics Laboratory Hub for processing for WGS. DNA and RNA will be extracted from tumour tissue and sequenced for mutational analysis and copy number aberrations. Where tissue and/or blood is available we would also like to perform analysis such RNA expression and methylation in collaboration with the CRUK Manchester Centre for Cancer Biomarker Sciences (MCCBS). STRECK blood samples will be processed for circulating tumour cell (CTC) enumeration and banked for future CTC analysis including genetic, transcriptome, proteomic and epigenetic changes. This will be undertaken within the Circulating Biomarker team within the MCCBS.

<u>Sample collection methods</u>

Blood sample collection for Foundation Medicine

- Foundation Medicine will supply sample selection kits
- Each kit will contain 2 x 'Roche Cell-Free DNA (cfDNA) blood collection tubes
- Blood specimens will **not** be returned, remaining blood or plasma not placed into testing is discarded in accordance with local practice.
- Typical turnaround time is approximately <2 weeks from receipt of specimen (see appendix 4)
- Further detail on the collection and shipment of these samples can be found in the sample collection kit

Tumour sample collection for Whole Genome Sequencing

- Detail on sample collection, preparation and shipment can be found in the laboratory manual
- Samples will be shipped to Manchester Genomics Hub (Manchester University NHS Foundation

DocuSign Envelope ID: BF6EC156-05BF-4243-A38A-AE415088BB25

Trust) for DNA extraction prior to Whole Genome Sequencing by Source Bioscience

Sample collection for CTCs and plasma for future analysis

- STRECK tubes will be supplied by CRUK Manchester Institute Centre for Cancer Biomarker Sciences (MCCBS)
- STRECK samples will be transferred to either the Christie Hospital or MCCBS for circulating tumour cell (CTC) or circulating nucleic acid processing.
- For CTC processing samples will require transfer and processing at room temperature to MCCBS within 72 hours, the remaining plasma will be stored for future analysis. For plasma processing for circulating nucleic acids samples must be received at either MCCBS at room temperature and processed within 4 days.
- Further details can be found in the laboratory manual

DocuSign Envelope ID: BF6EC156-05BF-4243-A38A-AE415088BB25

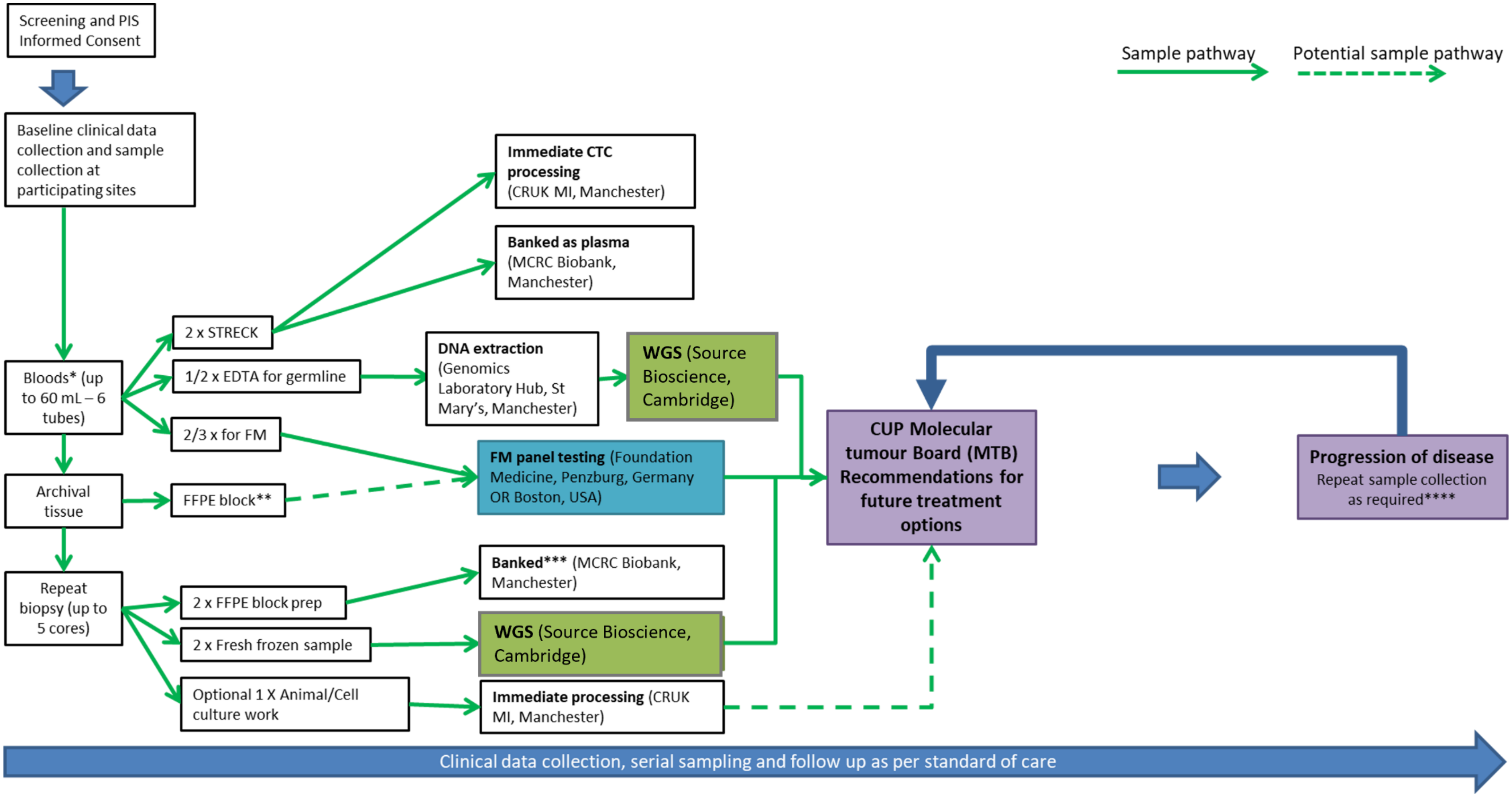

Figure 4: Sample pathway flow for CUP-COMP

*One tube of blood is approximately 10mls.

**FFPE blocks will be sent for FM panel testing if a fresh biopsy is not safe to undertake and/or if FM bloods tests are cancelled by the supplier for quality control

reasons. The quality of FFPE blocks should be reviewed by a pathologist before being sent for testing as detailed in the lab manual.

***Extra FFPE blocks will be banked at the Christie NHS Foundation Trust

****Repeat FM blood collection available.

CRUK MI: Cancer Research UK Manchester Institute

FM: Foundation Medicine

PIS: Patient information sheet

mL: millilitres

WGS: whole genome sequencing

Shipping locations are listed in the accompanying lab manual.

6.3 *Christie Site Only* – Retrospective Cohort of Archival Tissue

Within the Christie Hospital we have access to archival tissue from CUP patients for those patients consented to the CUP biobank. However, there is a cohort of CUP patients since 2013 that have been discussed at The Christie MDT and treated but are now deceased. These samples, now surplus to clinical need, provide a unique cohort of patients that have been through thorough pathological review and MDT. These samples are held within the Christie pathology laboratory, or referring trust, but do not have consent given by patients for use of tissue for research purposes and obtaining retrospective consent is not feasible. For these patients we wish to retrospectively molecular profile the archival tissue to inform on molecular heterogeneity of this cohort and produce data that may aid treatment decision algorithms for prospective patients. This dataset will also be used as a retrospective comparison dataset and used for research purposes to answer wider biological questions regarding CUP.

Cases with consent through MCRC CUP biobank (Reference:18_ALCO_01):
A cohort of patients undergoing follow up at The Christie hospital have prospectively provided informed consent for storage of archival tissue through Manchester Cancer Research Centre (MCRC) Biobank and this has ethical approval for use of these samples retrospectively.

Cases without consent:
Archival tissue of deceased patients, that is now surplus to requirements, is held within The Christie NHS Foundation Trust, or within the referring hospitals pathology departments. Samples originally taken from other trusts will have permission from original holder for use of surplus material prior to transfer to research team. Those samples identified as being returned to original Trust will be sought for permission and transfer to Christie pathology laboratory prior to anonymisation and transfer to research team.

Due to the poor survival of patients with CUP (<10% survival at 5 years, median survival of 6-9 months) it will be highly impracticable to approach patients, or their relatives, for permission to use tissue and clinical data for research. The results of any research performed will not have implications for the individual deceased patients, and data will be anonymised and therefore not be linked back to individual patients. Molecular characterisation of the tumour will only report somatic mutations and therefore no germline/heritable mutation detection will be reported.

The use of tissue prior to September 2006 from which consent regulations of Human Tissue Act (HTA) is exempt, is unfortunately not appropriate for this research. CUP UK national guidelines were introduced in 2010, initiating robust national diagnosis and pathological guidelines of these patients after the HTA came into effect. Tissue obtained before 2010 would therefore have had variable diagnostic pathological review and a true diagnosis of CUP as is classified today would be uncertain.

Experience of the Phase I team at The Christie have found patients extremely willing to have archival 'left over' tumour samples used for research purposes in the hope of improving knowledge and future treatments. The use of tissue alone without clinical data is ultimately of less value to future patients.

Identification and anonymisation of samples:
Cases of CUP from deceased individuals treated at The Christie Hospital since 2010 will be identified by the clinical team, and those with sufficient 'surplus' archival tissue remaining will be selected for study. Clinical demographic and outcome data will be obtained by the clinical team and anonymised coded tissue samples will be directed to collaborators for molecular profiling.

Collection of clinical data:
Clinical data for these patients will be obtained by authorised members of the clinical team in line with CUP-COMP protocol. Patients will be identified and collated from electronic case records including The Christie EPR, pathology and MDT databases. Data will then be anonymised and coded appropriately by a member of the clinical team, therefore clinical information will be non-identifiable to researchers. Anonymised data will be held securely within the CUP-COMP data lake in accordance with information governance regulations, and shared as appropriate and as per data transfer agreements between the CUP-COMP collaborators. It is proposed that linkage of clinical data to tissue samples holds no risk to participants. Anonymisation will occur by members of clinical team with legitimate access to patients' medical records.

Analysis of archival tissue 'FFPE': In line with CUP-COMP protocol, archival tissue will be sent for analysis by Foundation Medicine.

7.0 Recruitment and Selection of Study Participants

Potential study participants will be identified and assessed through discussions at local CUP MDTs.

7.1 Informed Consent

All potential study participants will be provided with a participant information sheet (PIS) and invited to voluntarily consent to participate in the study. It will be made clear to the potential study participant that consent is voluntary and can be withdrawn without giving reasons and without prejudicing their ongoing/future care.

Potential study participants will be given adequate time (at least 24 hours) to consider participation in the study before consent is elicited. When all queries have been addressed and the research team is confident

that the participant understands the study and all requirements participants will be consented to participate.

Patients will have the option to opt out of receiving any genetic results that may have significance for biological family members, or that are not related to their cancer but may have an impact on them.

Consent will be taken by a researcher who is appropriately trained and who has been delegated by the PI to undertake this activity (and this is clearly documented on the delegation log). No study-related activities can be undertaken prior to consent.

The original, signed copy of the participant information sheet and consent form(s) will be retained in the Investigator Site File, with a copy in the participant notes. A copy will also be given to the participant.

After consent has been obtained, the participant will be formally assessed for eligibility for the study at the screening visit against inclusion and exclusion criteria of the study protocol.

7.2 Participant Selection Criteria

All study participants must have a histological confirmed diagnosis of CUP based on the clinical, radiological and pathological review at a local or regional CUP MDT and the 2015 ESMO Clinical Practice Guidelines for CUP (Fizazi *et al.*, 2015). Favourable and unfavourable-risk CUP subsets are eligible. The population to be included in this study corresponds to patients with CUP of epithelial origin for whom a likely tissue of origin cannot be determined. All tests/examinations performed to ascertain the diagnosis of CUP and exclude a tumour of known origin (following ESMO guidelines) will be available and entered onto the study clinical research form (CRF). Any primary origin suggested by the clinical presentation or pathology work-up should be formally excluded prior to confirming a CUP. Participants will have undergone these procedures prior to the study as part of their routine care.

The diagnosis of CUP according to the 2015 ESMO guidelines should have included:

- Thorough physical examination (including e.g. head and neck, pelvic and breast examination)
- Basic blood analyses
- Computed tomography (CT) scans of thorax, abdomen and pelvis

In certain cases:

- Mammography in women
- Breast MRI in women with axillary adenocarcinoma
- Endoscopies guided by sign-, symptom- or laboratory abnormalities

- Whole-body FDG–PET/CT suggested for patients with cervical adenopathies from CUP and those with single CUP metastasis

Acceptable histology will include:

- Adenocarcinoma
- Poorly differentiated adenocarcinoma or carcinoma
- Squamous cell carcinoma

Histology and immunohistochemical profiles (per ESMO guidelines) that are not included above can be included but **MUST** be discussed with the Chief Investigator. Examples include non-epithelial cancer, neuroendocrine tumours and sarcoma.

All participants must meet all of the inclusion criteria and none of the exclusion criteria for this study at the time of registration.

7.3 Participant Inclusion Criteria

Participants are eligible to be included in the study only if **all** of the following criteria apply:

1. Aged 16 years or over
2. Written informed consent according to ICH/GCP and national regulations
3. ECOG Performance status 0-2
4. Confirmed diagnosis of CUP as per the ESMO guidelines (described above). Patients must have;
    a. The local pathology reports confirming compatibility with CUP diagnosis and the associated slides used for the diagnosis
    b. Discussion at a local CUP MDT confirming diagnosis
5. Accessible tumour that can be safely biopsied using radiological techniques. Biopsy may be undertaken as standard of care (surplus tissue sample to be used for this protocol), or maximum of one fresh biopsy specifically for purposes of the protocol. Subjects with inaccessible tumours for biopsy specimens but with a confirmed CUP diagnosis, may be enrolled without a biopsy upon consultation and agreement by the sponsor
6. Availability of archival tumour sample, slides and histological report
7. Willingness to provide blood samples on up to three occasions during the course of the study

7.4 Participant Exclusion Criteria

Participants are excluded from the study if **any** of the following criteria apply:

1. Patient with an immunohistochemistry profile that provides a definitive clinical indication of a primary cancer with a specific treatment
2. Known HIV, Hepatitis B, C positive, or COVID-19 positive, due to the difficulties in handling high-risk specimens
3. Patients who are unable to provide fully informed written consent
4. Presence of any medical, psychological, familial or sociological condition that, in the investigator's opinion, will hamper compliance with the study protocol and follow-up schedule
5. Bleeding diathesis (patients' on anticoagulation are permitted to enter the trial if anticoagulation can be safely managed to enable fresh tumour biopsies and blood sampling)

6. Conditions in which research biopsies or blood sampling may increase risk of complications for the patients and/or investigator

7.5 Study Payment

Participants will not receive any payment, nor be compensated for travel expenses related to the study.

7.6 Participant Registration

Participants who have consented to participate will be assigned a unique ID code which will be used throughout their participation in the study.

The ID code will be generated by individual sites using a pre-defined numbering system as follows:

CUP101-0001

Site = 101

Patient ID = 0001

CUP201-0001

Site = 201

Patient ID = 0001

This will be communicated to participating sites by the Project Manager.

The master list will be stored in a secure location and access will be restricted to authorised personnel. Please refer to sponsor SOP: R&D006.000 (Handling research data).

7.7 Discontinuation of Participants

Specific reasons for discontinuing a participant from study procedures are:

- Investigator decision
- Oversight committee recommendation/ decision
- Safety reasons
- Severe non-compliance to protocol as judged by the investigator and/or sponsor
- Participant's decision to withdraw (see section 7.8)
- Completed visits up to the End of Trial time point

7.8 Withdrawal of Participants

If at any time a participant expresses a wish to withdraw consent for ongoing study participation the following procedures will be observed:

1. Withdrawal of consent will be clearly documented in the study documentation and the study participant's medical record.
2. No further clinical data will be collected from the study participant. However, existing clinical data held will be retained and may still be used in future research by researchers who have a legitimate interest in the study data.
3. Samples will be retained and included in analyses unless the participant requests them to be destroyed. Samples not already processed/analysed will be destroyed upon request, samples already resulted will remain in the trial data set. It is the responsibility of the participating sites' research team to ensure that participant's wishes are met.
4. The study participant's privacy will be respected and preserved.

If a participant consents to the study but subsequently becomes unable to give consent they will be withdrawn from the study and no further information or samples will be collected. Previously obtained data and samples will still be used in the study.

8.0 Statistics and Data Analysis.

8.1 Sample Size

This study will recruit participants until 16th February 2023. We expect around 100-140 patients to consent prospectively over this time period across the seven sites open to recruitment. Each site will initially be given 20 slots to allocate, with any further slots allocated upon agreement with sponsor and CI. The nature of CUP and likelihood of many different tumour types being represented justifies as large a sample size as possible, however we are also only able to obtain funding for molecular testing, by both tumour (WGS and targeted sequencing) and ctDNA, for the patient numbers quoted. We anticipate some patients may not have adequate amounts of tissue available for the WGS analysis but they would not need to be replaced as we expect to have ctDNA results for all patients. In these patients we may also be able to perform targeted sequencing from their archival specimens, where there is enough tissue left for this analysis.

8.2 Recruitment Rate and Interim Analysis

There are seven recruiting centres for this study, spread geographically across the UK. From the study start date we will recruit participants until 16th February 2023 and aim to recruit 100-140 patients during this time period. We anticipate that there will be a 15-20% screen failure rate. Therefore the total number of patients for full molecular analysis will be approximately 110-120 patients. Each centre will be allocated 20 patient slots for recruitment. The centres will attend a monthly teleconference to discuss recruitment and new consents. We anticipate approximately 3-4 patients will consent each month (1 patient per site every other month). If some sites are not recruiting the numbers of patients anticipated other sites may be allocated extra slots. There is no planned interim analysis. Further external funding could be applied for at the end of this study dependent on the results.

9.0 Data Management

Clinical data for patients will be obtained by authorised and appropriately trained members of the clinical research teams at participating centres. Patients will be identified and collated from electronic case records from the patients NHS Trust. Data will then be pseudo -anonymised and coded appropriately by a member of the clinical team and therefore clinical information will be rendered non-identifiable for researchers. It is proposed that linkage of clinical data to tissue samples holds no risk to participants. Pseudo-anonymisation will be carried out by members of clinical team with legitimate access to patients medical records.

Samples will be pseudo-anonymised to researchers. Local and national procedures and policies in place will be applied to all personal data to ensure confidentiality and compliance with data protection act.

Mandatory clinical data that must be available;

- DOB
- Gender
- Ethnicity
- Date of onset of symptoms
- Date of referral to secondary care
- Date of diagnosis
- Date of biopsy

- Site of biopsy
- Disease sites/burden of disease
- Height/Weight
- Past medical history/Co-morbidities
- Medications
- Family history of cancer
- Smoking History
- Histology, including cellularity of tissue and immunohistochemical (IHC) analysis
- Pseudo-anonymised pathology report
- Pseudo-anonymised radiological report (CT scan or PET/CTscan)
- Blood parameters, to include Full blood count, Albumin, C-reactive protein, Liver function tests, Adjusted calcium, Blood glucose, Lactate dehydrogenase and tumour markers where performed
- Treatment type (Surgery/Radiotherapy/Chemotherapy/Other) and dates
- Date of treatment
- Date of progression
- Outcomes (date of progression/date of death)

Such data will only be used to facilitate clinical and translational research and will be stored in secure computers and will be protected by data confidentiality legislation.

Genomic data confidentiality: DNA samples will be evaluated by sequencing in order to identify “actionable” aberrations and to provide a molecular profile of individual cancer specimens. Additional analyses may be performed on tumour or circulating biomarkers to identify potentially actionable molecular aberrations or biomarkers relevant to the use of immunotherapy treatment. All samples will be pseudo-anonymised and the link between the samples ID and patients’ identifiable data will be stored in secure, password-protected computers. Clinical data will be handled according to the Data Protection Act 2018 and in accordance with the principles of ICH-GCP. The sequencing database will initially be separate from the clinical/sample database and access to one database will not provide automatic access to the other. In the global joint endeavour to understand the molecular basis of cancer, secondary research use maximises scientific utility and promotes the achievement of public benefit. Patients will be asked to consent to sharing of their anonymised data.

Sequencing results and disclosure: We are establishing a multi-disciplinary CUP Molecular Tumour Board (MTB) with expertise in medical oncology, experimental therapeutics and genomics. The sequencing result

(and/or supporting molecular profiling analyses) of each patient will be reviewed by the CUP MTB meetings who will provide oversight on the nature, clinical significance and the relevance of the sequencing results and will inform the local CUP team of any "actionable" mutations/aberrations, which could be useful to direct selection of a targeted therapy treatment for that patient.

Data analysis methods

ConcR data analysis: Patients recruited into the trial will be split into an analysis cohort (n= 95) and validation cohort (n=25), stratified by treatment, to ensure that the validation cohort has sufficient numbers of patients treated by each drug.

The molecular data collected from the WGS and Foundation One Liquid Biopsy will be used to build a subclonal breakdown of each patient's tumour, and the response of each subclone to treatment will be predicted by a dynamical model parameterised by the mutational composition of the subclone and other measured variables. These models will be parameterised by data from the analysis cohort from CUP- COMP and other data source, for example TCGA and GDSC. A validation cohort of 25 patients will be separated from the dataset.

9.1 Case Report Forms

Participating sites are responsible for maintaining source data for their study participants and for transcribing this onto a study-specific paper CRF which will be provided to sites. The Principal Investigator is responsible for ensuring the accuracy, completeness and legibility of the data recorded in the CRFs. Only the investigator and authorised personnel who have signed the delegation log should record or amend data in the CRFs. The paper CRF will be sent to the CUP-COMP project manager at The Christie NHS Foundation Trust where the data will be transcribed onto an electronic password controlled spreadsheet.

9.2 Data Storage

Participant's notes, completed paper CRFs and study files will be held in a secure storage area with limited access within The Christie NHS Foundation Trust and other participating NHS sites.

Computers used to collate data will have limited access measures via usernames and passwords.

9.3 Access to Data

Direct access will be granted to authorised representatives from the Sponsor, host institution and the regulatory authorities to permit trial-related audits and inspections. Informed consent will be obtained to cover this activity.

Aggregated pseudo-anonymised data will be made accessible to Roche, ConcR and Innovate UK. Once the project is completed and data has been published anonymised data will be made available to broader communities in the UK and globally to help drive innovation and research in CUP.

9.4 Confidentiality

All Investigators, collaborators and site staff involved with the study must comply with the requirements of The Data Protection Act 2018 and General Data Protection Regulations 2018 with regard to the collection, storage, processing and disclosure of personal information and will uphold the Act's core principles.

Any personal data recorded will be regarded as confidential, and any information which would allow individual participants to be identified will not be released in to the public domain.

9.5 Archiving

Essential documents will be maintained securely stored and access restricted to authorised personnel. Essential documents are documents that individually and collectively permit evaluation of the conduct of the study and substantiate the quality of the data collected.

Essential documents, including personal data will be stored at an approved external storage facility for a minimum of 5 years after the end of the study.

At the point where it is decided that the study documentation is no longer required, the sponsor will authorise the destruction of all essential documents related to the study.

10.0 Administrative and Regulatory Details

10.1 Study Sponsor

The Christie NHS Foundation Trust will act as the sponsor for this study. Delegated responsibilities will be assigned to the Chief Investigator and the CUP-COMP research team to manage the study on behalf of the sponsor.

10.2 Peer Review

This protocol has undergone high-quality peer review by members of The Christie NHS Foundation Trust Experimental Cancer Medicine Team, and external experts from F. Hoffmann-La Roche and ConcR. It was also sent for external peer review.

10.3 Funding Body

Innovate UK will act as primary the funder for this study. Some funds will also be provided by F. Hoffmann-La Roche and ConcR Funding for the virtual molecular tumour board has been provided by CUP Foundation – Jo's Friends.

This study is has been costed in accordance with DH guidelines: Attributing the costs of health & social care Research & Development (AcoRD).

The study will be submitted for adoption on the NIHR portfolio providing access to service support costs.

10.4 Ethics Approval

No participants will be entered onto the study before ethical approval has been confirmed. The study protocol has received the favourable opinion of the HRA Ethics Committee Yorkshire and The Humber – Sheffield Research Ethics Committee for the study protocol, informed consent forms and other relevant documents.

All participating sites must undergo site specific assessment of capacity and capability prior to their participation in the study. All investigators and key study personnel will be appropriately trained in Good Clinical Practice.

The study will be conducted in accordance with, but not limited to, the Human Rights Act 1998, The Data Protection Act 2018, General Data Protection Regulations 2018, Freedom of Information Act 2000, the Human Tissue Act 2004, and the UK Policy framework for Health and Social Care research as amended from time to time.

Where participants agree to take part in the study, they will be informed of how data are recorded, collected, stored and processed and may be transferred to other countries, in accordance with The General Data Protection Regulation (GDPR) (EU) 2016/679. As the sponsor of the study is a non-commercial organisation the legal basis for the handling and processing of data is 'task in the public interest.'

Annual progress reports will be submitted to REC within 30 days of the anniversary date on which the favourable opinion was given, and annually until the study is declared ended.

10.5 Participant and Public Involvement

Patient representatives from the CUP foundation Jo's Friends have been involved in aspects of trial design, documentation review and dissemination of results, and have contributed to the development of the protocol and patient information documents.

10.6 Study Monitoring

The study will be monitored by the sponsor, in accordance with the trial risk assessment through the completion and review of Sponsor Oversight Activity Reports (SOARs), Annual Progress Reports and Trial Management Oversight Committees. The study will be subject to The Christie Research division's audit

programme. In addition the study will be monitored and reviewed on a quarterly basis to ensure it is progressing well and in line with the Innovate UK funding competition rules.

10.7 Protocol Compliance

The study protocol must be adhered to at all times. Intentional deviations from the protocol are not permitted under any circumstances.

The research team will record technical/minor deviations in the study records. Such deviations will include isolated issues that do not put participants at risk of harm nor risk credibility of data; e.g. a single missed visit window.

The sponsor will be notified of other deviations significantly impacting the risk profile of the study or significantly interrupting study services or supplies via: the-christie.sponsoredresearch@ nhs.net. The Chief Investigator should also be notified.

Identified or suspected serious breaches of the study protocol or principles of GCP will be notified to the Sponsor's Operational Director of Research or Quality Manager via: 0161 918 7572 or the-christie.breaches@nhs.net

10.8 Safety Reporting

10.8.1 Terms and Definitions

10.8.1.1 Adverse Events

An Adverse Event (AE) is defined as any untoward medical occurrence in a participant who has undergone a research procedure, including occurrences which are not necessarily caused by or related to that procedure.

10.8.1.2 Serious Adverse Events

A Serious Adverse Event (SAE) is defined as an untoward occurrence that:

(a) Results in death;

(b) Is life-threatening;

(c) Requires hospitalisation or prolongation of existing hospitalisation;

(d) Results in persistent or significant disability or incapacity;

(e) Consists of a congenital anomaly or birth defect; or

(f) Is otherwise considered medically significant by the investigator

10.8.2 Notes on Adverse Event Inclusions and Exclusions

Planned hospitalisations as part of tumour tissue acquisition will not qualify as SAEs. Adverse events or hospitalizations secondary to other causes as judged by investigators like the anticancer treatment the patient may be receiving will not be considered an SAE for this study.

Serious adverse events related to study procedures will be reported by study personnel using the adverse event reporting form within 24 hours and evaluated by one of the study Co-Investigators. Reported SAEs will be followed up until resolution.

10.8.3 Evaluating Adverse Events

Any adverse event identified by a participating site will documented by study personnel using the adverse event report form.

The AE will be assessed for seriousness, causality and expectedness by the principal investigator or a delegated co-investigator at the relevant site.

Adverse events which meet the criteria to be reported as an SAE will be submitted to the CI/ delegate and the sponsor (the-christie.safety@nhs.net) within 24 hours of sites becoming aware of the event. Upon receipt, the Chief Investigator will review the event and the following will be considered:

- Whether there is a causal link with the study intervention
- Whether the event is or is not expected

The outcome of the assessment will be documented and filed in the trial master file.

10.8.4 Reporting of Serious Adverse Events

An SAE occurring to a research participant will be reported to the REC where in the opinion of the Chief Investigator the event was:

- “Related” – that is, it resulted from administration of any of the research procedures, and
- “Unexpected” – that is, the type of event is not listed in the protocol as an expected occurrence

Reports of related and unexpected SAEs will be submitted to the REC within 15 days of the Chief Investigator becoming aware of the event, using the SAE report form for non-CTIMPs published on the HRA website.

A copy of the complete report and the REC acknowledgement will be forwarded to the sponsor via email to the-christie.safety@nhs.net

10.9 Study Oversight

Study oversight will be managed by the following committees:

1) A Trial Management Group (TMG) will be established and will include those responsible for the day to day management of the study including the Chief Investigator, Co-investigators and study managers as detailed in the key contacts section of this protocol. The TMG will meet monthly to review recruitment and ensure the safety of study participants.

2) A Trial Steering Group (TSG) will be established to monitor ongoing progress of the trial. The purpose of these meetings will be to discuss the overall results and scientific merit of the study to date, highlight any areas of concern and/or issues, review resource and ongoing capacity and capability, and review any amendments. The TSG will consist of the Chief Investigator and Study Manager, and representatives from Roche, ConcR, Durham University, Innovate UK, Source Bioscience, Cancer Research UK Manchester Institute and the Sponsor. This group will meet quarterly.

3) A molecular tumour board (MTB) will be established to review sequencing results on a bi-weekly basis. Please see section 9.0 for detail.

10.10 Protocol Amendments

DocuSign Envelope ID: BF6EC156-05BF-4243-A38A-AE415088BB25

Any changes in research activity will require an amendment and will be initiated by the CI. Proposed changes must be submitted in writing to the sponsor. The amendment will be categorised as substantial or non-substantial.

Any required changes to the documents that supported the original ethical approval will be submitted as an amendment to the appropriate ethical and regulatory authorities by the research team, following the UK process for handling amendments. Substantial amendments will not be implemented until REC and HRA, if applicable, grant approval.

The research team will notify participating sites of all amendments. In accordance with the HRA amendments process, it is assumed that a participating site will implement the amendment unless communication to the contrary is received within 30 days of submission of the amendment to participating sites.

An Urgent Safety Measure (USM) may be put in place with immediate effect without gaining prior authorisation in order to protect participants from any immediate hazard to their health and safety. A discussion will take place within REC to agree timelines for the amendment submission. This discussion will be documented within the study file.

The research team will maintain an amendment history log to ensure that the most recent version of the protocol and supporting documents are used at all times.

10.11 Liability and Indemnity

As the sponsor is an NHS organisation the NHS indemnity scheme will apply in the event of harm to participants arising from the management of the study.

The University of Manchester's insurance and indemnity will apply in the event of harm to the participants arising from the design of the study.

Participating sties will be liable for clinical negligence and other negligent harm to participants taking part in the study and covered by the duty of care owed to them by the sites concerned. For participating sites which are part of the NHS, the NHS indemnity scheme will also apply.

10.12 End of Study Notification

The end of study notification will be submitted in writing to the REC within 90 days of the date of termination of the study as defined in section 5.4.

In the event that the study is terminated early, the notification will be submitted to REC within 15 days.

10.13 Study Summary Report

The study team and Chief Investigator are responsible for compiling and submitting the final study report to the sponsor and REC within one year of study closure.

10.14 Publication Policy

The data arising from the study will belong to the sponsor, The Christie NHS Foundation Trust. The Operational Director for Research will act as the data custodian for this study.

The main study results will be presented at congresses and published in a peer reviewed journal, on behalf of all coinvestigators and collaborators. Participants will be given the option to request a summary of the study results should they so wish.

The Publishing Party shall ensure that the contributions of the other relevant Parties, and Roche, are acknowledged in accordance with good academic practice. Prior to submission of publications or any other dissemination of the results, the Sponsor shall invite Roche and ConcR to review the draft publication for factual accuracy in relation to the FMI services and ConcR algorithm to ensure that Confidential Information is not disclosed and to ascertain whether the publication contains any patentable information.

The sponsor shall remove from the publication or presentation, any trade secret, propriety or Confidential Information disclosed by Roche or ConcR to the Trust or belonging to Roche. Scientific information regarding effectiveness of IMP(s), their side effects or adverse events that may appear as a result of their use in the concerned indication shall never be considered confidential.

11.0 References

Cancer Research UK (2020) *Carcinoma of Unknown Primary statistics*. Available at: https://www.cancerresearchuk.org/health-professional/cancer-statistics/statistics-by-cancer-type/cancer-of-unknown-primary#heading-Zero (Accessed: 11 June 2020).

Conway, A.-M. *et al.* (2019) 'Molecular characterisation and liquid biomarkers in Carcinoma of Unknown Primary (CUP): taking the "U" out of "CUP"', *British Journal of Cancer*, 120(2), pp. 141–153. doi: 10.1038/s41416-018-0332-2.

Culine, S. *et al.* (2003) 'Cisplatin in Combination With Either Gemcitabine or Irinotecan in Carcinomas of Unknown Primary Site: Results of a Randomized Phase II Study—Trial for the French Study Group on Carcinomas of Unknown Primary (GEFCAPI 01)', *Journal of Clinical Oncology*, 21(18), pp. 3479–3482. doi: 10.1200/JCO.2003.12.104.

Dahlman, K. B. *et al.* (2012) 'BRAF L597 Mutations in Melanoma Are Associated with Sensitivity to MEK Inhibitors', *Cancer Discovery*, 2(9), pp. 791–797. doi: 10.1158/2159-8290.CD-12-0097.

Drilon, A. (2019) 'TRK inhibitors in TRK fusion-positive cancers', *Annals of Oncology*, 30, pp. viii23–viii30. doi: 10.1093/annonc/mdz282.

Fizazi, K. *et al.* (2015) 'Cancers of unknown primary site: ESMO Clinical Practice Guidelines for diagnosis, treatment and follow-up', *Annals of Oncology*, 26, pp. v133–v138. doi: 10.1093/annonc/mdv305.

Fizazi, K. *et al.* (2019) 'A phase III trial of empiric chemotherapy with cisplatin and gemcitabine or systemic treatment tailored by molecular gene expression analysis in patients with carcinomas of an unknown primary (CUP) site (GEFCAPI 04)', *Annals of Oncology*, 30, p. v851. doi: 10.1093/annonc/mdz394.

Golfinopoulos, V. *et al.* (2009) 'Comparative survival with diverse chemotherapy regimens for cancer of unknown primary site: Multiple-treatments meta-analysis', *Cancer Treatment Reviews*, 35(7), pp. 570–573. doi: 10.1016/j.ctrv.2009.05.005.

Grigoriu, B., Berghmans, T. and Meert, A.-P. (2015) 'Management of EGFR mutated nonsmall cell lung

DocuSign Envelope ID: BF6EC156-05BF-4243-A38A-AE415088BB25

carcinoma patients', *European Respiratory Journal*, 45(4), pp. 1132–1141. doi: 10.1183/09031936.00156614.

Gross-Goupil, M. *et al.* (2012) 'Cisplatin alone or combined with gemcitabine in carcinomas of unknown primary: Results of the randomised GEFCAPI 02 trial', *European Journal of Cancer*, 48(5), pp. 721–727. doi: 10.1016/j.ejca.2012.01.011.

Hainsworth, J. D. *et al.* (2010) 'Paclitaxel/Carboplatin/Etoposide Versus Gemcitabine/Irinotecan in the First-Line Treatment of Patients With Carcinoma of Unknown Primary Site', *The Cancer Journal*, 16(1), pp. 70–75. doi: 10.1097/PPO.0b013e3181c6aa89.

Hayashi, H. *et al.* (2019) 'Randomized Phase II Trial Comparing Site-Specific Treatment Based on Gene Expression Profiling With Carboplatin and Paclitaxel for Patients With Cancer of Unknown Primary Site', *Journal of Clinical Oncology*, 37(7), pp. 570–579. doi: 10.1200/JCO.18.00771.

Kato, S. *et al.* (2017) 'Utility of Genomic Analysis In Circulating Tumor DNA from Patients with Carcinoma of Unknown Primary', *Cancer Research*, 77(16), pp. 4238–4246. doi: 10.1158/0008-5472.CAN-17-0628.

Ko, J. *et al.* (2018) 'Machine learning to detect signatures of disease in liquid biopsies – a user's guide', *Lab on a Chip*, 18(3), pp. 395–405. doi: 10.1039/C7LC00955K.

Lu, X. *et al.* (2018) 'Targeting EGFR L858R/T790M and EGFR L858R/T790M/C797S resistance mutations in NSCLC: Current developments in medicinal chemistry', *Medicinal Research Reviews*, 38(5), pp. 1550–1581. doi: 10.1002/med.21488.

Massard, C., Loriot, Y. and Fizazi, K. (2011) 'Carcinomas of an unknown primary origin—diagnosis and treatment', *Nature Reviews Clinical Oncology*, 8(12), pp. 701–710. doi: 10.1038/nrclinonc.2011.158.

Pavlidis, N. and Fizazi, K. (2009) 'Carcinoma of unknown primary (CUP)', *Critical Reviews in Oncology/Hematology*, 69(3), pp. 271–278. doi: 10.1016/j.critrevonc.2008.09.005.

Potie, N. *et al.* (2019) 'On the Need of Interpretability for Biomedical Applications: Using Fuzzy Models for Lung Cancer Prediction with Liquid Biopsy', in *2019 IEEE International Conference on Fuzzy Systems (FUZZ-IEEE)*. IEEE, pp. 1–6. doi: 10.1109/FUZZ-IEEE.2019.8858976.

DocuSign Envelope ID: BF6EC156-05BF-4243-A38A-AE415088BB25

Ross, J. S. *et al.* (2015) 'Comprehensive Genomic Profiling of Carcinoma of Unknown Primary Site', *JAMA Oncology*, 1(1), p. 40. doi: 10.1001/jamaoncol.2014.216.

Shaw, A. T. *et al.* (2014) 'Crizotinib in ROS1 -Rearranged Non–Small-Cell Lung Cancer', *New England Journal of Medicine*, 371(21), pp. 1963–1971. doi: 10.1056/NEJMoa1406766.

Tanizaki, J. *et al.* (2020) 'NivoCUP: An open-label phase II study on the efficacy of nivolumab in cancer of unknown primary.', *Journal of Clinical Oncology*, 38(15_suppl), pp. 106–106. doi: 10.1200/JCO.2020.38.15_suppl.106.

Appendix 1. Summary of 2015 ESMO clinical practice guidelines for CUP (taken from Fizazi *et al.* 2015)

CUP diagnosis should include:

- Thorough physical examination (including e.g. head and neck, pelvic and breast examination)
- Basic blood analyses
- Computed tomography (CT) scans of thorax, abdomen and pelvis

In certain cases:

- Mammography in women
- Breast MRI in women with axillary adenocarcinoma
- Endoscopies guided by sign-, symptom- or laboratory abnormalities
- Whole-body FDG–PET/CT suggested for patients with cervical adenopathies from CUP and those with single CUP metastasis

Acceptable histology will include:

- Adenocarcinoma
- Poorly differentiated adenocarcinoma or carcinoma
- Squamous cell carcinoma

Histology and immunohistochemical profiles (per ESMO guidelines) that are not included above can be included but **MUST** be discussed with the Chief Investigator. Examples include non-epithelial cancer, neuroendocrine tumours and sarcoma.

If there is suspicion of a primary cancer, yet following the above examinations a primary cannot be found, treatment would be recommended based on known available site specific therapies for the proposed cancer type.

Where there is no strong suspicion of a primary site, but a specific subset of CUP can be identified, specific treatment would be recommended (see figure 2, page 15).

Patients for which no specific subset of CUP can be identified, including those with favourable or poor prognosis, can be considered for enrolment into the study.

Appendix 2: Serious adverse event report form

Serious Adverse Event

CUP-COMP CFTSp188

The Christie
Research and Innovation

**Study and subject details**	**For Project Team use only - SAE ID no:**
Date: ☐☐ / ☐☐ / ☐☐☐☐	Notification type: ☐ Initial ☐ FU1 ☐ FU2 ☐ FU3 ☐ Other
Site	Participant no.

**SAE details and concomitant treatment**	
SAE category*	SAE start date/time
*Categories: 1) Fatal 2) Life threatening 3) Involved or prolonged hospitalisation 4) Disability or incapacity 5) Other (describe in SAE details)	
SAE medical term	
SAE details (provide a brief description of the event, grade and treatment given to treat SAE etc)	

Date or duration of treatment	Outcome+	+ Outcome categories:
		1) Resolved 2) Resolve with sequelae<br>3) Ongoing, no therapy 4) Ongoing, therapy 5) Death
Relevant diagnostic tests		

Concomitant treatment(s)<br>(exclude those used to treat SAE) OR attach relevant CRF pages	Date(s) of administration
Relevant patient medical history OR attach CRF	

Reporter details			
Reporter name	Reporter email	Reporter phone no.	Reporter fax no.

**PI assessment of SAE causality and expectedness** (to be completed by the local PI or appropriately qualified delegate only)			
Could the trial intervention(s) have reasonably caused the SAE?		☐ No<br>☐ Yes	If the two right-hand boxes are checked, event is an unexpected and related event and will be reported to REC.
If caused by the trial intervention(s), is the nature, severity, seriousness or outcome of the SAE consistent with the reference safety information? i.e. can it be expected from the intervention(s)?		☐ Yes<br>☐ No<br>☐ NA	
If there is more than one intervention, specify which is likely to have caused the SAE			
PI signature		Date	

**CI assessment of SAE causality and expectedness** (to be completed by the CI or appropriately qualified delegate only)			
Could the trial intervention(s) have reasonably caused the SAE?		☐ No ☐ Yes	If the two right-hand boxes are checked, event is an unexpected and related event and will be reported to REC.
If caused by the trial intervention(s), is the nature, severity, seriousness or outcome of the SAE consistent with the reference safety information? i.e. can it be expected from the intervention?		☐ Yes ☐ No<br>☐ NA	
If there is more than one intervention, specify which is likely to have caused the SAE			
CI signature		Date	

Notes for completion:

For initial notifications: Assessment by the CI is not required on the initial report and this and further details may be sent later. Please ensure that any patient-identifying information is redacted before providing medical history or other requested information.

Follow-up reports: Follow-up information should be sent as soon as possible after the initial report. Apart from trial / SAE identifiers and reporter contact details, there is no need to duplicate information previously sent. Please complete only those fields with the additional information.

It is not necessary to notify those SAEs indicated in the protocol as not requiring immediate reporting.

Errors or corrections should be scored through with a single line and the correct entry clearly written with the corrector's initials and the date. Correction fluid or tape is not permitted.

Please store original copies in the Investigator Site File.

DocuSign Envelope ID: BF6EC156-05BF-4243-A38A-AE415088BB25
